# β‐Adrenergic Stimulation Induces Histone Deacetylase 5 (HDAC5) Nuclear Accumulation in Cardiomyocytes by B55α‐PP2A‐Mediated Dephosphorylation

**DOI:** 10.1161/JAHA.116.004861

**Published:** 2017-03-25

**Authors:** Kate L. Weeks, Antonella Ranieri, Agnieszka Karaś, Bianca C. Bernardo, Alexandra S. Ashcroft, Chris Molenaar, Julie R. McMullen, Metin Avkiran

**Affiliations:** ^1^ Cardiovascular Division King's College London British Heart Foundation Centre of Research Excellence The Rayne Institute St Thomas' Hospital London United Kingdom; ^2^ Baker Heart and Diabetes Institute Melbourne Australia

**Keywords:** adrenergic stimulation, B55α, confocal imaging, histone deacetylase 5, hypertrophy/remodeling, microscopy, phosphatase, phosphorylation, PP2A, three dimensional, β‐adrenergic signaling, Basic Science Research, Cell Signalling/Signal Transduction, Mechanisms, Myocardial Biology, Hypertrophy

## Abstract

**Background:**

Class IIa histone deacetylase (HDAC) isoforms such as HDAC5 are critical signal‐responsive repressors of maladaptive cardiomyocyte hypertrophy, through nuclear interactions with transcription factors including myocyte enhancer factor‐2. β‐Adrenoceptor (β‐AR) stimulation, a signal of fundamental importance in regulating cardiac function, has been proposed to induce both phosphorylation‐independent nuclear export and phosphorylation‐dependent nuclear accumulation of cardiomyocyte HDAC5. The relative importance of phosphorylation at Ser259/Ser498 versus Ser279 in HDAC5 regulation is also controversial. We aimed to determine the impact of β‐AR stimulation on the phosphorylation, localization, and function of cardiomyocyte HDAC5 and delineate underlying molecular mechanisms.

**Methods and Results:**

A novel 3‐dimensional confocal microscopy method that objectively quantifies the whole‐cell nuclear/cytoplasmic distribution of green fluorescent protein tagged HDAC5 revealed the β‐AR agonist isoproterenol to induce β_1_‐AR‐mediated and protein kinase A‐dependent HDAC5 nuclear accumulation in adult rat cardiomyocytes, which was accompanied by dephosphorylation at Ser259/279/498. Mutation of Ser259/Ser498 to Ala promoted HDAC5 nuclear accumulation and myocyte enhancer factor‐2 inhibition, whereas Ser279 ablation had no such effect and did not block isoproterenol‐induced nuclear accumulation. Inhibition of the Ser/Thr phosphatase PP2A blocked isoproterenol‐induced HDAC5 dephosphorylation. Co‐immunoprecipitation revealed a specific interaction of HDAC5 with the PP2A targeting subunit B55α, as well as catalytic and scaffolding subunits, which increased >3‐fold with isoproterenol. Knockdown of B55α in neonatal cardiomyocytes attenuated isoproterenol‐induced HDAC5 dephosphorylation.

**Conclusions:**

β‐AR stimulation induces HDAC5 nuclear accumulation in cardiomyocytes by a mechanism that is protein kinase A‐dependent but requires B55α‐PP2A‐mediated dephosphorylation of Ser259/Ser498 rather than protein kinase A‐mediated phosphorylation of Ser279.

## Introduction

Histone deacetylase (HDAC) 5 is a class IIa HDAC isoform that is an important negative regulator of maladaptive cardiomyocyte hypertrophy and pathological cardiac remodeling.^1^ Unlike class I HDACs, which repress gene transcription by altering chromatin structure via the deacetylation of histones, class IIa HDACs regulate transcription via the direct repression of transcription factors, such as members of the myocyte enhancer factor‐2 (MEF2) family,^2^ and by recruiting epigenetic regulators (such as class I HDACs and histone methyltransferases) to DNA in large, multiprotein repressor complexes.^3^, ^4^, ^5^, ^6^ Nuclear export of class IIa HDACs leads to dissociation of these repressor complexes, which promotes gene transcription by permitting the recruitment of transcriptional activators (such as histone acetyltransferases and histone demethylases) and the binding of transcription factors to gene promoter/enhancer regions.^5^ Thus, subcellular localization is a key determinant of class IIa HDAC function.

HDAC5 contains at least 17 phospho‐acceptor residues^7^ and altered phosphorylation is a key posttranslational mechanism regulating its nuclear/cytoplasmic distribution (see recent reviews^8^, ^9^). Of particular interest are S259 and S498 flanking a nuclear localization sequence, whose phosphorylation alters HDAC5 localization in cardiomyocytes in response to biologically relevant neurohormonal stimuli. Stimulation of G_q_ protein‐coupled receptors (G_q_PCRs), such as the α_1_‐adrenergic receptor or the endothelin‐1 receptor, induces the phosphorylation of S259/S498 by HDAC kinases, such as protein kinase D^10^ and Ca^2+^/calmodulin‐dependent protein kinase II.^11^, ^12^ This in turn triggers a conformational change that exposes a C‐terminal nuclear export sequence, leading to CRM1‐dependent nuclear export.^11^, ^13^, ^14^ Phosphorylated S259 and S498 also provide docking sites for 14‐3‐3 proteins, which mask the nuclear localization sequence, preventing re‐entry into the nucleus.^15^, ^16^ In contrast to G_q_PCR agonists, our recent work indicates that stimulation of the G_s_ protein‐coupled β‐adrenergic receptor (β‐AR) with isoproterenol reduces HDAC5 phosphorylation at S259/S498 in cardiomyocytes, but may trigger HDAC5 nuclear export through a phosphorylation‐independent mechanism.^17^

In recent years, phosphorylation of S279 has also been proposed as an important mechanism regulating HDAC5 subcellular localization in cardiomyocytes. S279 can be directly phosphorylated by protein kinase A (PKA) in in vitro kinase assays, and interventions that activate β‐AR/PKA signaling block G_q_PCR‐mediated nuclear export of HDAC5 in neonatal rat ventricular myocytes (NRVM).^18^ Furthermore, data from the use of phospho‐mimetic (S279D) and nonphosphorylatable (S279A) mutants of HDAC5 support a role for S279 phosphorylation in driving the nuclear retention of HDAC5 in this cell type.^18^ Broadly similar findings were reported subsequently from studies using adult rabbit ventricular myocytes.^19^ However, it is important to note that, to date, there is no evidence for increased HDAC5 phosphorylation at S279 occurring in response to β‐AR‐mediated PKA activation in cardiomyocytes.

Given that β‐AR signaling is a key therapeutic target in heart failure because of its critical role in the regulation of cardiac structure and function,^20^, ^21^ we set out to address the following principal aims: (1) To definitively determine the impact of β‐AR/PKA signaling on the phosphorylation, subcellular localization, and function of HDAC5 in adult cardiomyocytes; (2) To delineate the relative importance of altered phosphorylation at S259/S498 (established nuclear export sites) versus S279 (putative nuclear import site) in regulating the subcellular distribution of HDAC5 in this cell type; and (3) To determine the molecular mechanisms responsible for altered phosphorylation at the key regulatory residues. Toward these aims, we developed a new imaging method that allows the comprehensive quantification of the whole‐cell nuclear/cytoplasmic distribution of green fluorescent protein (GFP) tagged HDAC5 in living adult rat ventricular myocytes (ARVM), utilizing fluorescent dyes for objective demarcation of the nuclear and cytoplasmic compartments in conjunction with 3‐dimensional confocal microscopy. Our data show that β‐AR stimulation induces the nuclear accumulation of HDAC5 through a mechanism that is mediated by the β_1_‐AR subtype and PKA activity. Furthermore, our data indicate that β‐AR stimulation induces PP2A‐mediated dephosphorylation of HDAC5 at both the S259/S498 and the S279 sites, and that reduced phosphorylation at the S259/S498 sites is the principal mechanism of β_1_‐AR/PKA/PP2A‐mediated nuclear accumulation. Finally, we show that β‐AR activation increases the interaction between HDAC5 and PP2A catalytic and scaffolding subunits, as well as the PP2A targeting subunit B55α selectively. Silencing of B55α in NRVM blocked isoproterenol‐induced dephosphorylation at the S259 site, suggesting that this subunit is required for PP2A‐mediated dephosphorylation of HDAC5 downstream of β‐AR activation in cardiomyocytes. These findings point to a role for B55α‐PP2A‐mediated HDAC5 dephosphorylation and nuclear accumulation in preventing MEF2‐dependent gene transcription during short‐term β‐AR stimulation.

## Methods

Detailed methodology is provided in Data S1. Data are presented as mean±SEM, unless otherwise stated in the figure legend. Normally distributed data sets were analyzed by unpaired *t* test, 1‐way ANOVA or 2‐way ANOVA as appropriate. Data sets that failed the D'Agostino and Pearson omnibus normality test were analyzed by Mann–Whitney test (2 groups) or Kruskal–Wallis 1‐way ANOVA by ranks (2 or more groups). ARVM experiments were performed in accordance with the Guidance on the Operation of Animals (Scientific Procedures) Act, 1986 (UK). NRVM experiments were conducted in accordance with the Australian Code for the Care and Use of Animals for Scientific Purposes (National Health & Medical Research Council of Australia, 8th Edition, 2013) and were approved by the Alfred Medical Research and Education Precinct's Animal Ethics Committee.

## Results

### β_1_‐AR/PKA Activation Induces Dephosphorylation of HDAC5 at S259/S498 and S279

To investigate the effects of β‐AR stimulation on the phosphorylation status of HDAC5 at S279, we made use of a recently described rabbit polyclonal antibody that detects phosphorylation of the conserved serine residue (S266) in HDAC4.^22^ As the phospho‐peptide sequence used to generate the anti‐pS266 HDAC4 antibody shares 100% amino acid identity with the pS279 motif in HDAC5 (Figure S1A), we predicted that this antibody would cross‐react with phosphorylated S279 in HDAC5. This was indeed the case, with the antibody detecting a protein of the correct molecular weight (≈150 kDa) in ARVM expressing wildtype (WT) GFP‐HDAC5 or GFP‐HDAC5 in which the S259 and S498 residues were mutated to alanine (S259/498A), but not in ARVM expressing GFP alone or a S279A mutant of GFP‐HDAC5 (Figure S1B). We also confirmed the phospho‐specificity of 2 commercially available antibodies (3443 from Cell Signaling and 47283 from Abcam) that detect pS259 or pS498 (Figure S1B; signals in ARVM expressing WT or S279A GFP‐HDAC5, but not in ARVM expressing S259/498A GFP‐HDAC5 or GFP alone).

**Figure 1:**
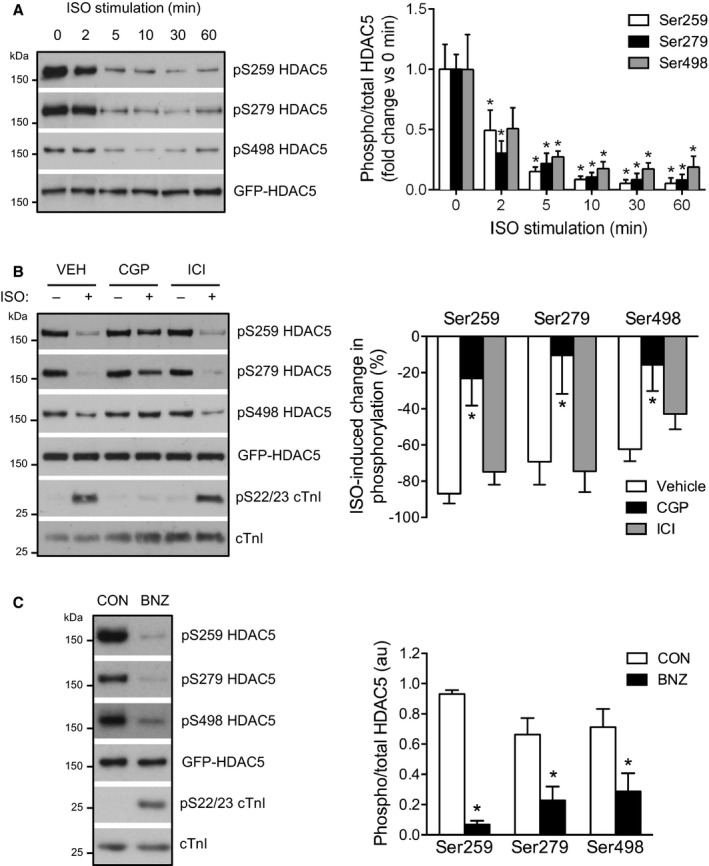
β_1_‐AR/PKA activation reduces HDAC5 phosphorylation. A, Time course of HDAC5 dephosphorylation by 10 nmol/L isoproterenol (ISO). Representative Western blots and grouped data from 4 independent experiments. Data obtained for each phosphorylation site were analyzed by 1‐way ANOVA followed by Dunnett's multiple comparisons tests. *P<0.05 vs 0 minutes. B, ARVM were treated with 10 nmol/L ISO or vehicle control (CON) for 10 minutes in the presence of 100 nmol/L CGP‐20712A (CGP; a β_1_‐AR‐selective antagonist), 100 nmol/L ICI 118,551 (ICI; a β_2_‐AR‐selective antagonist), or vehicle (VEH). Representative Western blots and grouped data from 6 independent experiments. One‐way ANOVA followed by Dunnett's multiple comparisons tests for each phosphorylation site. *P<0.05 vs VEH. C, ARVM were treated with 500 μmol/L N^6^‐benzoyl cAMP (BNZ) to selectively activate PKA or vehicle control (CON) for 30 minutes. Representative Western blots and grouped data from 5 independent experiments (n=4 for S498 site). Unpaired *t* test for each phosphorylation site. *P<0.05 vs CON. β_1_‐AR indicates β‐adrenergic receptor; ARVM, adult rat ventricular myocytes; BNZ, N^6^‐benzoyl cAMP; cTnI, cardiac troponin I; HDAC5, histone deacetylase 5; PKA, protein kinase A.

Consistent with our previous report,^17^ stimulation of ARVM expressing WT GFP‐HDAC5 with 10 nmol/L isoproterenol resulted in a rapid reduction in phosphorylation of S259 and S498 (Figure 1A). The maximum response occurred after ≈10 minutes and was sustained for at least 60 minutes. Interestingly, the proposed PKA target site, S279, responded in a similar manner, exhibiting marked dephosphorylation (Figure 1A). To investigate whether these effects occurred downstream of the β_1_‐ or β_2_‐AR, ARVM were treated with CGP‐20712A (a β_1_‐AR‐selective antagonist) or ICI 118,551 (a β_2_‐AR‐selective antagonist) for 10 minutes prior to isoproterenol stimulation. CGP‐20712A blocked both isoproterenol‐induced dephosphorylation of HDAC5 at all 3 sites and isoproterenol‐induced phosphorylation of cardiac troponin I (cTnI) at S22/S23, established PKA target sites that are phosphorylated downstream of β_1_‐AR stimulation (Figure 1B). Treating cells with ICI 118,551 at an identical concentration (CGP‐20712A and ICI 118,551 have comparable *K*_i_ values for their target β‐AR subtypes^23^) had no effect on isoproterenol‐induced HDAC5 dephosphorylation or cTnI phosphorylation (Figure 1B). These data indicate that HDAC5 dephosphorylation following isoproterenol stimulation occurs downstream of the β_1_‐AR subtype.

Next, we investigated the role of the cAMP‐activated effectors PKA and Epac in this β_1_‐AR‐mediated response. Directly activating PKA with the PKA‐selective cAMP analogue N^6^‐benzoyl cAMP induced a similar degree of cTnI phosphorylation at S22/S23 as 10 nmol/L isoproterenol, indicating comparable PKA activation (Figure S2A). N^6^‐benzoyl cAMP treatment also resulted in robust dephosphorylation of HDAC5 at all 3 sites, similar to that observed with isoproterenol (Figure 1C). In contrast, an identical concentration of the Epac‐selective cAMP analogue, 8‐CPT‐2′‐O‐Me‐cAMP (CPT), had no effect on cTnI or HDAC5 phosphorylation status (Figure S2B and S2C). These findings indicate that PKA activation alone is sufficient to induce HDAC5 dephosphorylation.

### β_1_‐AR/PKA Signaling Induces Nuclear Accumulation of HDAC5

β‐AR stimulation with isoproterenol has been reported to induce both nuclear export^17^ and nuclear accumulation^19^ of GFP‐HDAC5 in adult cardiomyocytes. To definitively delineate the impact of β‐AR stimulation on GFP‐HDAC5 distribution in this cell type, we established a new imaging method for assessing the nuclear and cytoplasmic contents of GFP‐HDAC5 in living ARVM. Previous methods for quantifying the relative abundance of GFP‐HDAC5 (and other GFP‐tagged proteins) in nuclear versus cytoplasmic compartments of adult cardiomyocytes have used either widefield fluorescence microscopy^17^, ^24^ or confocal microscopy of a single z‐section^19^, ^25^ to image GFP fluorescence within selected regions before and after exposure to various stimuli. Each of these studies utilized the GFP fluorescence signal to select and focus the cells for imaging, and to define nuclear and cytoplasmic regions of interest within which the average fluorescence intensity (*F*) was quantified. In preliminary experiments using z‐stack confocal microscopy, we found that the focal plane had a significant impact on the measured fluorescence intensity, particularly within the nuclear compartment (*F*_nuc_; Figure S3A and S3B) but also within the cytoplasmic compartment (*F*_cyto_; Figure S3C). *F*_nuc_ also often varied markedly between different nuclei in individual binucleated cells (Figure S3B).

**Figure 2:**
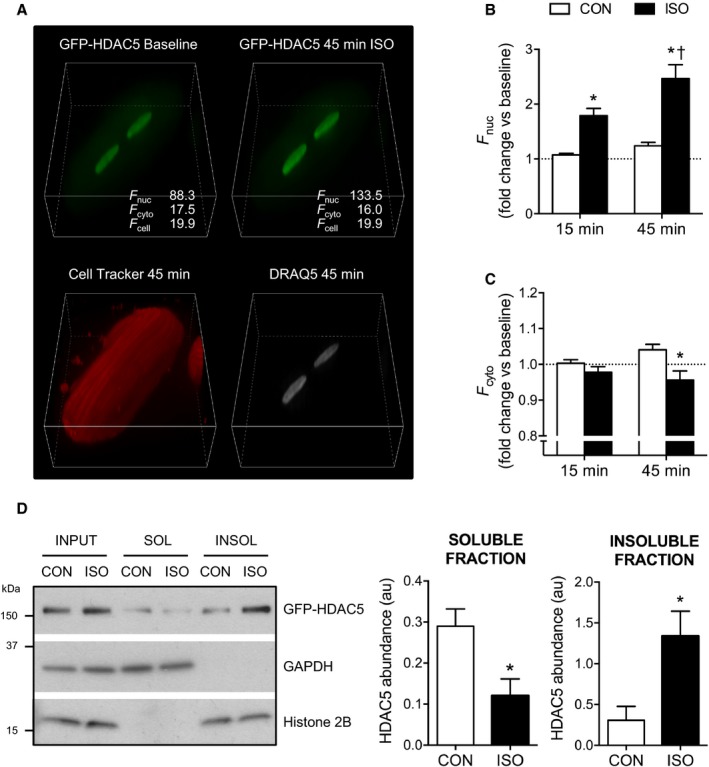
β‐adrenergic stimulation induces nuclear accumulation of GFP‐HDAC5. A, 3‐Dimensional projections of an ARVM expressing GFP‐HDAC5 at baseline and 45 minutes after the addition of 10 nmol/L isoproterenol (ISO). Z‐stacks (25 z‐sections at 1.5‐μm intervals) were acquired as described in the Methods section. The fluorescent signals from the Cell Tracker Orange and DRAQ5 dyes were used to define the cytoplasmic (cyto) and nuclear (nuc) volumes for objective quantification of the average fluorescence intensity (*F*) arising from the GFP‐HDAC5 signal within those compartments. B and C, Quantification of *F*_nuc_ and *F*_cyto_ following treatment with 10 nmol/L ISO or vehicle control (CON) for 15 to 45 minutes. All measurements obtained at the 15‐ and 45‐minute time points were normalized to the baseline value for each cell (denoted by the dotted line). Two‐way repeated‐measures ANOVA followed by Sidak's multiple comparisons tests. *P<0.05 vs CON at the same time point. ^†^ *P*<0.05 vs the same treatment group at the 15‐minute time point. n=17 to 22 cells from 3 hearts per group. D, ARVM were fractionated into Triton X‐100‐soluble (SOL) and ‐insoluble (INSOL) fractions via centrifugation following treatment with 10 nmol/L ISO or CON for 45 minutes. Representative Western blots show the distribution of GFP‐HDAC5, GAPDH (cytosolic protein), and histone 2B (nuclear protein). Quantitative data show GFP‐HDAC5 abundance in soluble and insoluble fractions from 5 independent experiments. In each experiment, the GFP‐HDAC5 signal in CON and ISO fractions was normalized to the respective input. Unpaired *t* tests. *P<0.05 vs CON. ARVM indicates adult rat ventricular myocytes; GFP, green fluorescent protein; HDAC5, histone deacetylase 5.

To eliminate the variability and potential subjectivity associated with sampling small regions of interest, and to account for differences in the focal plane between cells or focal drift over the time course of an experiment, we used spinning disk confocal microscopy to acquire z‐stacks (1.5‐μm steps spanning 36 μm) to capture information from the whole cell. Cells were imaged at baseline and at 15 and 45 minutes after the addition of vehicle control or isoproterenol, to allow the assessment of the temporal effects of each treatment. Additionally, myocytes were labeled with Cell Tracker Orange the day before imaging, and nuclei were labeled with DRAQ5 10 minutes before the final time point. Images subsequently obtained during excitation of the Cell Tracker Orange and DRAQ5 fluorophores with a laser beam at 561 or 640 nm, respectively, were used to objectively delineate the cytoplasmic and nuclear compartments (Figure 2A). This allowed quantification of the GFP fluorescence signal in each compartment (*F*_cyto_ and *F*_nuc_) in an unbiased manner and calculation of the nuclear/cytoplasmic *F* ratio (*F*_nuc_/*F*_cyto_) at each time point. For time course experiments, *F*_nuc_/*F*_cyto_ ratios obtained at the 15‐ and 45‐minute time points were normalized to the baseline value for each cell, to account for differences in the basal distribution of GFP‐HDAC5 between cells. Strict inclusion criteria were adhered to during the selection of cells for imaging and the postimaging quantification of signals was conducted in a treatment‐blinded manner (see Methods for further details).

**Figure 3:**
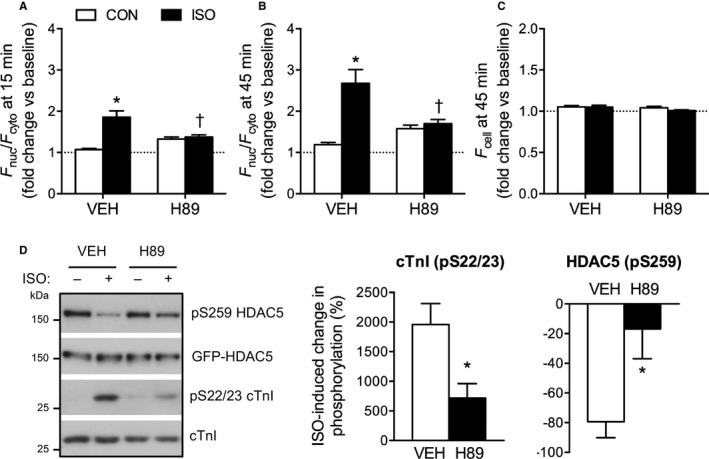
ISO‐induced dephosphorylation and nuclear accumulation of HDAC5 is PKA‐dependent. A and B, Quantification of the nuclear/cytoplasmic fluorescence ratio (*F*_nuc_/*F*_cyto_) in ARVM treated with 10 nmol/L ISO or CON in the presence of 10 μmol/L H89 (PKA inhibitor) or vehicle (VEH). *F*_nuc_/*F*_cyto_ ratios were normalized to the baseline value obtained for each cell to account for differences in the basal distribution of GFP‐HDAC5 between cells. Two‐way ANOVA followed by Tukey's multiple comparisons test. *P<0.05 vs CON within the same pretreatment group (VEH/H89). ^†^ *P*<0.05 vs VEH within the same treatment group (CON/ISO). n=17 to 30 cells from 3 hearts per group. C, Quantification of *F*_cell_ at the 45‐minute time point, normalized to baseline. No significant differences were detected by 2‐way ANOVA. D, ARVM were treated with 10 nmol/L ISO or CON in the presence of 10 μmol/L H89 or vehicle (VEH). Representative Western blots and grouped data from 5 independent experiments. Unpaired *t* test. *P<0.05 vs VEH. ARVM indicates adult rat ventricular myocytes; CON, control; cTnI, cardiac troponin I; GFP, green fluorescent protein; HDAC5, histone deacetylase 5; ISO, isoproterenol; PKA, protein kinase A.

The application of the above methodology revealed isoproterenol to induce significant nuclear accumulation of GFP‐HDAC5 (Figure 2A), quantified as an increase in *F*_nuc_ (Figure 2B) and a decrease in *F*_cyto_ (Figure 2C) and a corresponding increase in the *F*_nuc_/*F*_cyto_ ratio (see vehicle‐treated control and isoproterenol groups, Figure 3A and 3B). Importantly, there was no change in the whole cell fluorescence (*F*_cell_) over the course of the experiment (Figure 3C). To validate our findings, we determined the distribution of GFP‐HDAC5 in ARVM by a fractionation approach. Briefly, ARVM were fractionated into Triton X‐100‐soluble and ‐insoluble fractions via centrifugation. Treatment with isoproterenol resulted in depletion of GFP‐HDAC5 from the soluble fraction, which contained cytosolic proteins such as GAPDH, and an enrichment of GFP‐HDAC5 in the insoluble fraction, which contained nuclear proteins such as histone 2B (Figure 2D). These data conclusively demonstrate that acute β‐adrenergic stimulation triggers the nuclear accumulation of GFP‐HDAC5 in adult cardiomyocytes.

Next, we investigated the importance of PKA in regulating this process. The isoproterenol‐induced nuclear accumulation of GFP‐HDAC5 was blocked by pretreatment with H89 (Figure 3A and 3B), an isoquinolinesulfonamide that suppresses PKA activity through competitive inhibition at the ATP binding site of the kinase.^26^ *F*_cell_ did not change significantly in response to isoproterenol and/or H89 treatment over the course of these experiments (Figure 3C). Biochemical analyses revealed such pretreatment with H89 to inhibit isoproterenol‐induced cTnI phosphorylation, confirming effective inhibition of PKA activity, as well as isoproterenol‐induced HDAC5 dephosphorylation (Figure 3D). Taken together, these findings indicate that PKA activity is necessary for both isoproterenol‐induced dephosphorylation and isoproterenol‐induced nuclear accumulation of HDAC5.

### S279 Phosphorylation Is Not Required for HDAC5 Nuclear Accumulation

**Figure 4:**
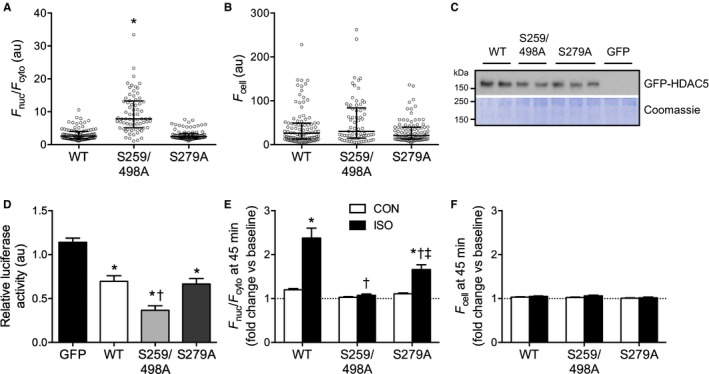
The phosphorylation status of S259/S498, and not S279, is the key determinant of GFP‐HDAC5 localization in ARVM. A and B, *F*_nuc_/*F*_cyto_ and *F*_cell_ for ARVM expressing wildtype (WT) or nonphosphorylatable variants (S259/498A, S279A) of GFP‐HDAC5. All individual data points, as well as the median and interquartile range, are shown. Kruskal‐Wallis 1‐way analysis of ranks followed by Dunn's multiple comparisons test. *P<0.05, n=69 to 98 cells from 3 to 6 hearts per group. C, Western blot showing comparable expression of GFP‐HDAC5 variants in ARVM. D, ARVM expressing GFP‐HDAC5 variants or GFP alone were cotransduced with adenoviruses encoding a MEF2‐luciferase reporter. ARVM were lysed and the luminescence readings from 25 μg protein were normalized to an internal control (ARVM expressing the luciferase reporter but not GFP/GFP‐HDAC5). One‐way ANOVA followed by Tukey's multiple comparisons test. *P<0.05 vs GFP, ^†^ *P*<0.05 vs WT. n=6 to 7 independently transduced samples from 2 hearts per group. E and F, ARVM expressing WT or nonphosphorylatable GFP‐HDAC5 variants were treated with 10 nmol/L isoproterenol (ISO) or vehicle control (CON) for 45 minutes. *F*_nuc_/*F*_cyto_ ratios and *F*_cell_ values were normalized to the baseline value obtained for each cell to account for differences in the basal distribution of GFP‐HDAC5 between cells. Two‐way ANOVA followed by Tukey's multiple comparisons test. *P<0.05 vs CON for the same GFP‐HDAC5 variant. ^†^ *P*<0.05 vs WT ISO, ^‡^ *P*<0.05 vs S259/498A ISO. n=17 to 40 cells from 3 hearts per group. ARVM indicates adult rat ventricular myocytes; GFP, green fluorescent protein; HDAC5, histone deacetylase 5; MEF, myocyte enhancing factor.

As noted earlier, it has been proposed that PKA‐mediated phosphorylation of S279 drives the nuclear import of HDAC5 following β‐AR activation.^19^ However, in our experiments, isoproterenol stimulation induced dephosphorylation of S279, as well as S259/S498 (Figure 1), while also triggering the nuclear accumulation of HDAC5 (Figures 2 and 3). To determine the relative importance of altered phosphorylation at S279 versus S259/S498 in regulating the subcellular distribution of HDAC5, we imaged cells expressing nonphosphorylatable mutants of GFP‐HDAC5 (S259/498A and S279A). Under basal conditions, S259/498A GFP‐HDAC5 was more nuclear than WT GFP‐HDAC5, whereas the S279A mutant displayed a similar subcellular distribution to WT GFP‐HDAC5 (Figure 4A). *F*_cell_ did not differ significantly between the groups (Figure 4B), consistent with the fact that we expressed the variants to similar levels (Figure 4C).

In complementary experiments, we also investigated the impact of expressing each GFP‐HDAC5 variant on MEF2 activity in ARVM co‐expressing a luciferase reporter under the transcriptional control of a MEF2‐sensitive promoter. MEF2 is a well‐characterized binding partner of HDAC5, and disruption of their interaction following phosphorylation‐dependent nuclear export of HDAC5 permits MEF2‐dependent gene transcription that promotes maladaptive cardiomyocyte hypertrophy.^13^, ^15^, ^27^ Relative to the heterologous expression of GFP alone, that of WT GFP‐HDAC5 repressed MEF2 activity (Figure 4D). Expression of the S259/498A variant, which showed greater nuclear accumulation (Figure 4A), led to a further reduction in MEF2 activity (Figure 4D). In contrast, expression of the S279A variant, which showed comparable subcellular distribution to the WT protein (Figure 4A), had a similar inhibitory effect on MEF2 activity (Figure 4D). These data suggest that the phosphorylation status of S279 does not have a significant impact on the subcellular distribution of HDAC5 or its function as a repressor of MEF2 transcriptional activity under basal conditions. On studying the impact of isoproterenol stimulation on the subcellular distribution of these HDAC5 variants, we found such stimulation to have no effect on S259/498A GFP‐HDAC5 localization, but to induce significant nuclear accumulation of S279A GFP‐HDAC5, albeit to a lesser extent than WT GFP‐HDAC5 (Figure 4E). Once again, there was no treatment effect on *F*_cell_ in any study group (Figure 4F). These findings indicate that the phosphorylation status of S259/S498 is the critical determinant of HDAC5 subcellular localization, and thereby HDAC5‐mediated MEF2 inhibition, and suggest that β_1_‐AR/PKA‐mediated dephosphorylation of these sites is responsible for isoproterenol‐induced HDAC5 nuclear accumulation. Our data also indicate that S279 phosphorylation is not necessary for isoproterenol‐induced HDAC5 nuclear import, firstly because no increase is observed in S279 phosphorylation of WT GFP‐HDAC5 over the time course during which its nuclear accumulation is observed (when, in fact, S279 becomes dephosphorylated) and secondly because isoproterenol stimulation induces significant nuclear accumulation of HDAC5 even when the S279 phosphorylation site has been ablated.

### PP2A Dephosphorylates HDAC5 Downstream of β‐AR Stimulation

**Figure 5:**
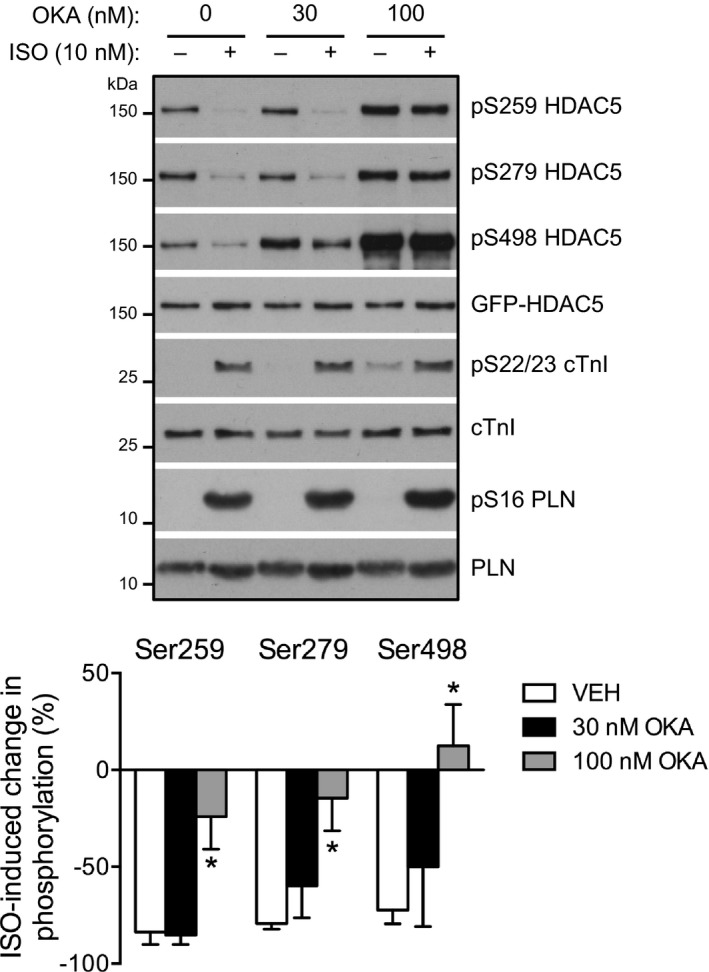
PP2A dephosphorylates HDAC5 downstream of β‐AR activation. ARVM were treated with 10 nmol/L isoproterenol (ISO) or vehicle control for 10 minutes in the presence of vehicle (VEH), 30 or 100 nmol/L okadaic acid (OKA). Representative Western blots and grouped data from 5 independent experiments. One‐way ANOVA followed by Dunnett's multiple comparisons tests for each phosphorylation site. *P<0.05 vs VEH. β‐AR indicates β‐adrenergic receptor; ARVM, adult rat ventricular myocytes; cTnI, cardiac troponin I; GFP, green fluorescent protein; HDAC5, histone deacetylase 5; PLN, phospholamban; PP2A, protein phosphatase 2A.

Given the apparent mechanistic importance of HDAC5 dephosphorylation at S259/S498 in facilitating β_1_‐AR/PKA‐mediated HDAC5 nuclear accumulation, we also explored the mechanism(s) by which such dephosphorylation might occur. Since isoproterenol‐induced dephosphorylation occurs very rapidly (within 2–5 minutes; Figure 1A), recruitment or activation of a protein phosphatase, rather than attenuated activity of an HDAC kinase, such as protein kinase D,^28^, ^29^ is likely to be involved. On the basis of previous studies in other cell types that have implicated PP2A activity as an important regulator of the phosphorylation and subcellular distribution of various class IIa HDAC isoforms^30^, ^31^, ^32^, ^33^ and work in HEK 293 cells that identified isoforms of all 3 subunits of the PP2A holoenzyme as binding partners of HDAC5,^7^ we hypothesized that PP2A may function as an HDAC5 phosphatase in ARVM. To test this, we treated cells with okadaic acid (OKA), a marine‐sponge toxin that inhibits the catalytic activity of both PP1 and PP2A family enzymes but exhibits higher affinity for the latter,^34^ prior to isoproterenol stimulation. Pretreatment with OKA at 100 nmol/L, but not 30 nmol/L, inhibited the isoproterenol‐induced dephosphorylation of HDAC5 at all 3 sites (Figure 5). OKA also increased the basal phosphorylation of cTnI at S22/S23, which are known PP2A substrates,^35^ with a similar concentration‐response profile. In contrast, OKA had no effect on the phosphorylation of phospholamban (PLN) at S16, which is a known PP1 substrate,^36^ indicating that 100 nmol/L OKA was sufficient and necessary to selectively inhibit PP2A activity in our experimental system. These findings suggest that PP2A activity is required for isoproterenol‐induced dephosphorylation of HDAC5.

### B55α Targets PP2A to HDAC5 for Dephosphorylation

PP2A family members are holoenzymes, each consisting of a scaffolding (A) subunit, a catalytic (C) subunit, and a regulatory (B) subunit, with substrate specificity conferred by the identity of the B subunit isoform.^37^ The B subunit isoform B55α (also known as PR55α; encoded by *PPP2R2A*) has been identified previously as a component of the PP2A complex that associates with heterologously expressed HDAC5 in HEK 293 cells.^7^ The same group also identified B55α as an interaction partner for HDAC5, but not any of 10 other human HDACs, in a proteomics screen performed in T cells heterologously expressing EGFP‐tagged HDAC isoforms.^38^ These findings prompted us to investigate whether B55α, which is expressed in the heart,^39^ might be responsible for targeting the PP2A holoenzyme to HDAC5 in ARVM.

**Figure 6:**
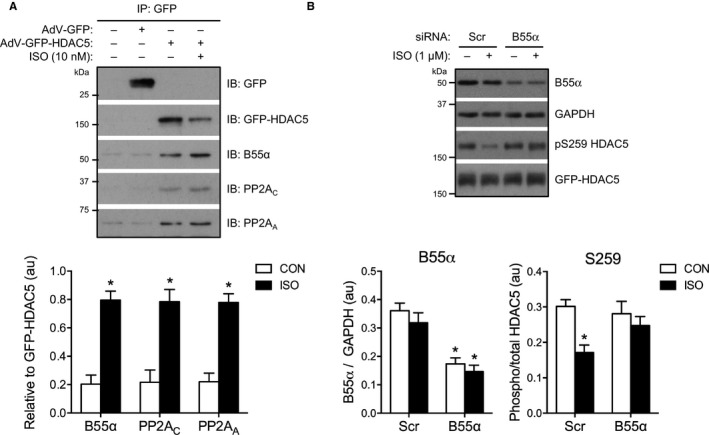
B55α targets PP2A to HDAC5 for dephosphorylation. A, ARVM expressing GFP or GFP‐HDAC5 were treated with 10 nmol/L isoproterenol (ISO) or vehicle control (CON) for 10 minutes. GFP/GFP‐HDAC5‐containing complexes were immunoprecipitated from cell lysates, separated by SDS‐PAGE and the resulting Western blots probed for B55α, PP2A_C_, and PP2A_A_. Representative Western blots and grouped data from 4 independent experiments. Unpaired *t* tests. *P<0.05 vs CON. B, NRVM expressing GFP‐HDAC5 were transfected with scrambled (Scr) or *PPP2R2A* (B55α) siRNAs for 48 hours prior to treatment with 1 μmol/L ISO or CON for 60 minutes. Representative Western blots and grouped data from 4 independent experiments. Two‐way ANOVA following by Sidak's multiple comparisons tests. For B55α: *P<0.05 vs Scr within the same treatment group (CON/ISO). For S259: *P<0.05 vs CON within the same siRNA group (Scr/B55α). AdV indicates adenovirus; ARVM, adult rat ventricular myocytes; GFP, green fluorescent protein; HDAC5, histone deacetylase 5; NRVM, neonatal rat ventricular myocytes; PP2A, protein phosphatase 2A.

B55α was readily detectable in ARVM, using a commercially available antibody (Figure S4A). To explore a potential HDAC5/B55α interaction, we immuno‐purified the GFP‐HDAC5 complex from ARVM following treatment with isoproterenol or vehicle control, and subjected it to SDS‐PAGE and immunoblot analysis. In pilot experiments we found that B55α, but not two other B subunit isoforms (B56α and B56δ), was associated with GFP‐HDAC5 in the absence and presence of isoproterenol stimulation (Figure S4B). No such association was observed with GFP (Figure S4B), indicating a specific interaction with HDAC5. Replicate experiments focusing on the potential interaction of HDAC5 with the B55α‐PP2A heterotrimeric holoenzyme revealed that not only B55α, but also the C and A subunits of PP2A, associated with HDAC5 (Figure 6A). Consistent with our fractionation experiments (Figure 2D), the amount of GFP‐HDAC5 that could be immunoprecipitated from the soluble cell lysate declined following isoproterenol stimulation (Figure 6A, top panel), presumably reflecting the accumulation of GFP‐HDAC5 in the nucleus, where it binds to DNA‐associated partners such as MEF2. Importantly, when normalized for the GFP‐HDAC5 content of the immunoprecipitated complex, the relative abundance of each of the components of the B55α‐PP2A holoenzyme within the complex increased >3‐fold following isoproterenol stimulation (Figure 6A, bottom panel). We confirmed that the interaction between B55α and HDAC5 also occurs with endogenous proteins by immunoprecipitating HDAC5 from cell lysates of NRVM which, relative to ARVM, exhibit more abundant expression of HDAC5. Consistent with our findings in ARVM expressing GFP‐HDAC5, B55α associated with endogenous HDAC5 in NRVM, and the interaction increased with isoproterenol (Figure S5). These findings indicate that activation of β‐AR signaling in cardiomyocytes promotes the association of B55α‐PP2A with HDAC5 and suggest that this may be a key mechanism underlying isoproterenol‐induced HDAC5 dephosphorylation. Recruitment of the PP2A holoenzyme to HDAC5 by B55α is likely to occur in the cytosol, as subcellular fractionation of ARVM revealed that B55α is present almost exclusively in the soluble fraction, and does not translocate to the insoluble fraction upon isoproterenol stimulation (Figure S6).

To assess the importance of B55α‐PP2A in mediating isoproterenol‐induced dephosphorylation of HDAC5, we designed an siRNA experiment to reduce the expression of B55α. As conventional transfection is ineffective in ARVM, and B55α targeting of HDAC5 appears to be conserved in NRVM (Figure S5), we introduced siRNAs targeting *PPP2R2A* transcripts into NRVM expressing GFP‐HDAC5. This resulted in a ≈50% to 55% reduction in B55α protein levels (Figure 6B). Treatment with 1 μmol/L isoproterenol for 1 hour caused a significant reduction in S259 phosphorylation that was abolished by B55α knockdown (Figure 6B), suggesting that B55α is required for PP2A‐mediated dephosphorylation of HDAC5 downstream of β‐AR stimulation. Isoproterenol had no effect on the phosphorylation of S279 or S498 in the absence or presence of B55α knockdown in these experiments (Figure S7).

### Phosphorylation‐Independent Regulation of B55α Downstream of β‐AR Activation

**Figure 7:**
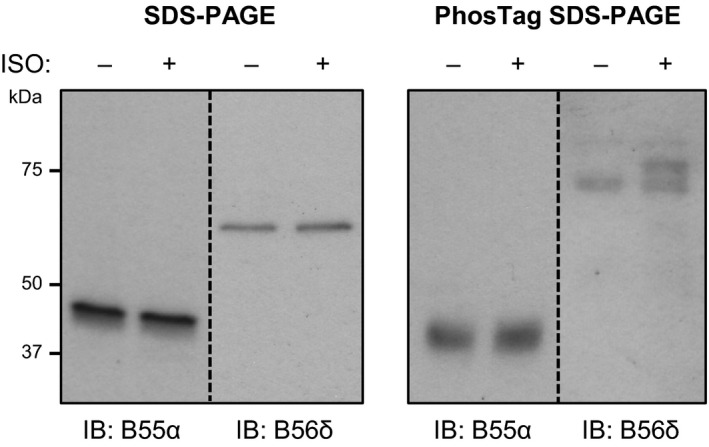
B55α is not phospho‐regulated downstream of β‐AR activation. Lysates from ARVM treated with vehicle control or 10 nmol/L isoproterenol (ISO) for 10 minutes were subjected to standard SDS‐PAGE or PhosTag phosphate‐affinity SDS‐PAGE. Duplicate sets of samples were run on the same gels for subsequent immunoblotting with antibodies against B55α and B56δ. Unlike B56δ, B55α is not phosphorylated in response to ISO treatment (multiple bands in ISO sample on PhosTag blot probed for B56δ; absence of multiple bands in ISO sample on PhosTag blot probed for B55α). β‐AR indicates β‐adrenergic receptor; ARVM, adult rat ventricular myocytes.

Since the function of some PP2A B subunit isoforms, such as B56δ, is regulated through PKA‐mediated phosphorylation,^40^ we also explored whether isoproterenol stimulation induces the phosphorylation of B55α in ARVM. To achieve this, we subjected protein samples from ARVM to PhosTag phosphate‐affinity SDS‐PAGE and immunoblot analysis.^41^ This revealed increased phosphorylation of B56δ, which is consistent with previous data from other cell types,^40^ but not B55α, in response to isoproterenol stimulation (Figure 7). Thus, isoproterenol‐induced targeting of B55α to HDAC5 in ARVM is unlikely to occur through increased phosphorylation of this B subunit.

## Discussion

The key findings of this study are that, in ARVM: (1) isoproterenol stimulation induces the nuclear accumulation of HDAC5, through a mechanism that requires PKA activity; (2) The key determinant of HDAC5 subcellular localization is the phosphorylation status of S259/S498, with loss of phosphorylation at these sites promoting nuclear accumulation and repression of MEF2; (3) The phosphorylation status of S279 plays a relatively insignificant role in regulating the nuclear versus cytoplasmic distribution of HDAC5; and (4) Dephosphorylation of HDAC5 following β‐AR activation requires PP2A activity. In addition, our data provide evidence that the regulatory subunit isoform B55α targets PP2A to HDAC5 downstream of β‐AR stimulation in cardiomyocytes, and is required for the dephosphorylation of HDAC5 at S259. Collectively, these findings may have important implications regarding the molecular mechanisms underlying cardiomyocyte responses to β‐AR stimulation in physiological and pathological settings.

Our findings concerning PP2A‐mediated dephosphorylation of HDAC5 add significantly to data from an earlier study in NRVM, which has shown that pretreatment with the PP2A inhibitor OKA inhibits the ability of isoproterenol stimulation to attenuate HDAC5 phosphorylation in response to G_q_PCR stimulation.^30^ Firstly, we show that β‐AR stimulation triggers HDAC5 dephosphorylation even in the absence of G_q_PCR stimulation. Secondly, we provide data indicating that, upon β‐AR stimulation, B55α is likely to target the PP2A holoenzyme to HDAC5 to facilitate its dephosphorylation at the key regulatory phospho‐acceptor residues. The mechanism by which β‐AR stimulation induces an increased B55α‐PP2A/HDAC5 association in cardiomyocytes requires further investigation. In this regard, recent crystallographic analyses of the B55α‐PP2A complex have defined not only the structure of the holoenzyme but also the likely binding interfaces between the B55α subunit and another phosphoprotein substrate of B55α‐PP2A, Tau.^42^ The pertinent data indicate that the likely binding site for Tau is a central groove on the top face of B55α and that a cluster of amino acids on one side of this groove play a critical role in binding.^42^ Importantly, individual mutations of such amino acids had a dramatic impact on the ability of the holoenzyme to bind to and dephosphorylate Tau.^42^ On the assumption that the interaction of B55α‐PP2A with HDAC5 occurs in a similar manner, posttranslational modification of certain residue(s) within the substrate‐binding surface of B55α may provide a mechanism through which β‐AR stimulation promotes B55α‐PP2A targeting to HDAC5, although our data from PhosTag phosphate affinity analysis suggest that isoproterenol stimulation does not induce a marked increase in B55α phosphorylation. Alternatively, posttranslational modification of B55α‐interacting residues within HDAC5 may play a role in this phenomenon. In that regard, Tau appears to possess two nonoverlapping peptide segments that are capable of binding to the B55α‐PP2A holoenzyme, which has been proposed to facilitate the dephosphorylation of hyperphosphorylated Tau that contains multiple phosphorylated Ser/Thr residues throughout its sequence.^42^ In analogy, although the focus of our present work and other related studies has been on the roles of 3 distinct phospho‐acceptor residues (S259, S279, and S498), proteomics analysis indicates that HDAC5 may contain at least 17 phosphorylated Ser/Thr residues,^7^ whose regulation and functional roles remain largely unknown.

**Figure 8:**
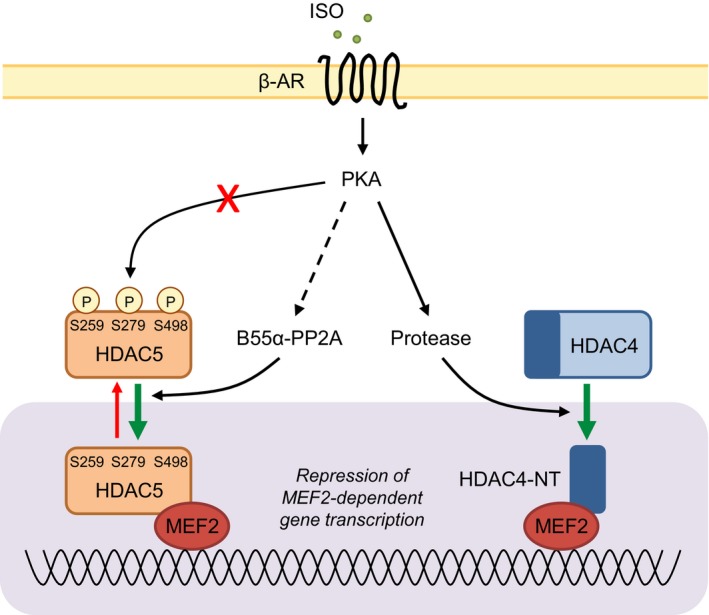
Working model for the regulation of class IIa histone deacetylases downstream of β‐AR activation in adult cardiomyocytes. Acute stimulation of β‐adrenergic receptors (β‐ARs) does not lead to nuclear accumulation of HDAC5 through PKA‐dependent phosphorylation of HDAC5 at S279, as previously proposed.^18^, ^19^ Rather, activation of β_1_‐ARs in adult cardiomyocytes leads to PP2A‐mediated dephosphorylation of HDAC5 at S259/S498 and S279, likely following the recruitment of PP2A to HDAC5 by the regulatory subunit B55α. Dephosphorylation of S259/S498 is the key driver of HDAC5 nuclear accumulation downstream of β_1_‐AR/PKA activation in ARVM. This mechanism may act in concert with the PKA‐dependent formation of an N‐terminal HDAC4 cleavage product (HDAC4‐NT)^43^ to repress MEF2‐dependent gene transcription in settings of acute/transient β‐adrenergic receptor stimulation. ARVM indicates adult rat ventricular myocytes; β‐AR, β‐adrenergic receptor; HDAC5, histone deacetylase 5; MEF2, myocyte enhancing factor‐2; PKA, protein kinase A; PP2A, protein phosphatase 2A.

Backs and colleagues^43^ have published convincing evidence that PKA activation in cardiomyocytes induces the generation of an N‐terminal cleavage product of another class IIa HDAC, HDAC4, and that this cleavage product (termed HDAC4‐NT) binds to and inhibits MEF2 in the nucleus. Based on these findings, Backs et al^43^ have proposed that the generation of HDAC4‐NT during repetitive transient sympathetic activation, as occurs during exercise training, would inhibit MEF2 activation and consequent pathological cardiac remodeling driven by MEF2‐regulated genes.^43^ They have also speculated that the therapeutic benefit afforded by β‐AR antagonists in human heart failure may arise, at least in part, through the attenuation of β‐AR desensitization and consequently greater HDAC4‐NT generation downstream of β‐AR stimulation.^43^ It is possible that the β‐AR/PKA/B55α‐PP2A‐mediated dephosphorylation and consequent nuclear accumulation of HDAC5 that we have uncovered in the present study provides a complementary protective mechanism against maladaptive cardiomyocyte hypertrophy and pathological cardiac remodeling, through the inhibition of MEF2 activity (Figure 8), although this requires further investigation. Of direct relevance to the potential protective effects of β‐AR‐mediated generation of HDAC4‐NT and dephosphorylation of HDAC5 are the earlier observations of Perrino et al,^44^ who reported that although exercise training and intermittent left ventricular pressure overload both induced comparable increases in circulating norepinephrine and epinephrine (the endogenous β‐AR agonists), only the latter stimulus led to β‐AR dysfunction and thereby produced a pathological cardiac phenotype. Determination of the physiological role of β‐AR/PKA/B55α‐PP2A‐mediated dephosphorylation and nuclear accumulation of HDAC5 requires the development of experimental approaches to specifically interfere with cardiac B55α and/or its targeting to HDAC5 upon β‐AR stimulation in an in vivo setting, which is a focus of our ongoing work. In this context, it is interesting to note recent evidence suggesting that B55α may dissociate from the core A/C dimer of PP2A in failing canine myocardium as a consequence of increased phosphorylation and/or reduced methylation of the C subunit,^39^ which, based on our findings, would be expected to lead to the attenuation of β‐AR/PKA/B55α‐PP2A‐mediated dephosphorylation and of the consequent nuclear accumulation of HDAC5.

The cellular imaging methodology that we have established and utilized in the present study provides the opportunity to objectively determine the nuclear versus cytoplasmic distribution of fluorescently tagged proteins in live cardiomyocytes, or indeed any other cell type, through the concomitant use of fluorescent dyes with distinct spectral properties (DRAQ5, which intercalates with DNA, and Cell Tracker Orange, which is retained in the cytoplasm) to demarcate the nuclear compartment and the remainder of the cellular volume. A key advantage of this methodology is the ability to monitor total fluorescence in each compartment across the entire cell, without the potential subjectivity that arises from the use of investigator‐selected regions of interest and/or z‐section, variations in both of which we have found to have a marked impact on the intensity of the GFP‐HDAC5 fluorescence signal. Application of this method has allowed us to definitively determine the impact of isoproterenol stimulation on HDAC5 nucleocytoplasmic shuttling in adult cardiomyocytes, and to interrogate the roles of the S259/S498 and S279 phosphorylation sites in regulating HDAC5 localization. Our findings regarding the impact of isoproterenol stimulation on the subcellular distribution of HDAC5 are consistent with the earlier reports of Ha et al,^18^ who studied NRVM stimulated with forskolin (which elevates cAMP by activating adenylyl cyclase), cAMP, or isoproterenol (1 μmol/L), and Chang et al,^19^ who studied adult rabbit ventricular myocytes stimulated with forskolin or isoproterenol (100 nmol/L), which both suggested that activation of the β‐AR/PKA pathway promotes the nuclear retention of HDAC5. Where our study differs markedly from such work is on the molecular mechanism(s) underlying the nuclear accumulation of HDAC5 in response to activation of the β‐AR/PKA pathway. Both Ha et al^18^ and Chang et al^19^ proposed PKA‐mediated phosphorylation of S279 (erroneously referred to as S280 in Ha et al^18^) to be the principal underlying mechanism. However, it is important to note that neither study provided direct evidence for such phosphorylation occurring following activation of the β‐AR/PKA pathway in cardiomyocytes, and their common proposed mechanism was based on observations made with mutated versions of HDAC5 carrying nonphosphorylatable (S279A) or phospho‐mimetic (S279D) substitutions at the pertinent site.^18^, ^19^ In the present study, we provide novel evidence from the use of a phospho‐specific antibody, which we have confirmed to specifically recognize HDAC5 phosphorylation at S279, that isoproterenol‐induced activation of the β‐AR/PKA pathway in ARVM produces no increase in the phosphorylation of this site. To the contrary, and in common with the S259/S498 sites (Haworth et al^17^ and the present study), phosphorylation of the S279 site declines rapidly and to a significant extent upon isoproterenol stimulation, and this dephosphorylation response is maintained for at least 60 minutes. Our findings are consistent with a previous study in striatal neurons, which reported reduced phosphorylation of all three sites following forskolin treatment.^33^ Notably, in our work, we used isoproterenol at a concentration of 10 nmol/L, which is 10‐ or 100‐fold lower than the concentrations used by Chang et al^19^ and Ha et al*,* 18 respectively. In ARVM, 10 nmol/L isoproterenol induces marked β‐AR‐mediated physiological responses, such as the potentiation of sarcomere shortening and intracellular [Ca^2+^] transient amplitude^29^, ^45^ and myofilament protein phosphorylation,^45^, ^46^ providing the rationale for our selection of this concentration in exploring the effects of isoproterenol on HDAC5 phosphorylation and localization. Determination of whether a different effect on HDAC5 phosphorylation at S279 might arise from the use of supra‐physiological concentrations of isoproterenol is beyond the scope of this study.

In our previous work utilizing widefield fluorescence microscopy and the quantification of fluorescence intensity in selected regions of interest, we reported the same concentration of isoproterenol (10 nmol/L) to induce GFP‐HDAC5 nuclear export in ARVM,^17^ which appears at odds with our current findings. Interestingly, in the course of the present work, we observed that while isoproterenol stimulation induced a decline in *F*_cyto_ and increases in *F*_nuc_ and *F*_nuc_/*F*_cyto_ across the whole cell without impacting on *F*_cell_ (Figures 2 and 3), it also led to the appearance of GFP‐HDAC5‐containing puncta in the cytoplasm of ≈60% of the cells that were imaged (Figure S8). These puncta were numerous and very bright relative to the GFP‐HDAC5 fluorescence in the surrounding cytoplasmic compartment (Figure S8). Furthermore, they did not arise from baseline mislocalization of GFP‐HDAC5 (Figure S9) and did not occur with time‐matched vehicle treatment or in ARVM expressing GFP alone. As widefield fluorescence microscopy also captures secondary fluorescence emitted from beyond the focal plane, the generation of such bright puncta and their impact on the measured *F*_cyto_ are likely to have contributed to the apparent isoproterenol‐induced decrease in *F*_nuc_/*F*_cyto_ in our previous study.^17^ The nature and potential biological significance of these isoproterenol‐induced GFP‐HDAC5‐containing puncta are currently unknown. Based on previous work in our laboratory^17^ and by others,^47^ however, it is possible that redox modification of reactive cysteine residues in HDAC5 may play a causal role in their appearance.

To conclude, in the present study, we describe a new method for studying the nuclear versus cytoplasmic localization of GFP‐HDAC5 in adult cardiomyocytes, which has allowed us to conclusively demonstrate that β‐AR stimulation induces the nuclear accumulation of HDAC5 through a mechanism that requires PKA activity. Furthermore, we show that dephosphorylation of S259/S498 in HDAC5 is the key mechanism that triggers nuclear accumulation, and that such dephosphorylation occurs downstream of the β_1_‐AR subtype and requires both PKA and PP2A activity, with the latter targeted to HDAC5 through the PP2A regulatory subunit B55α. These findings reveal new avenues of investigation toward a better understanding of the molecular mechanisms through which altered sympathetic activity and β‐AR signaling impact on cardiac phenotype, particularly in the context of cardiac remodeling.

## Sources of Funding

This research was supported by the British Heart Foundation (Project Grant PG/12/48/29638 and Centre of Research Excellence Award RE/13/2/30182) and in part by the Victorian Government's Operational Infrastructure Support Program. Weeks is supported by an Overseas Research Fellowship from the Heart Foundation of Australia (O12M6802). Bernardo is supported by an Alice Baker and Eleanor Shaw Fellowship from the Baker Foundation (Melbourne, Australia).

## Disclosures

None.

## Supporting information


**Data S1.** Supplemental methods.
**Figure S1.** Specificity of antibodies for pS259, pS279, and pS498 in HDAC5. A, Region of human HDAC4 used to generate an antibody against phosphorylated S266 (Walkinshaw et al, 2013) and sequence alignment showing 100% amino acid identity with human HDAC5. The conserved PKA phosphorylation site (S266 in HDAC4; S279 in HDAC5) is underlined. B, Cell lysates from ARVM expressing GFP, wildtype (WT) GFP‐HDAC5, or nonphosphorylatable (S259/498A; S279A) variants of GFP‐HDAC5 were separated by SDS‐PAGE and analyzed by Western blotting. The pS266 HDAC4 polyclonal rabbit antibody detected a band of the correct molecular weight in cells expressing WT and S259/498A GFP‐HDAC5, but not in cells expressing S279A GFP‐HDAC5 or GFP alone, demonstrating cross‐reactivity with pS279 in HDAC5. Similarly, commercially available antibodies for pS259 (Cell Signaling 3443) and pS498 (Abcam 47283) detected bands of the correct molecular weights in cells expressing WT and S279A GFP‐HDAC5, but not in cells expressing S259/498A GFP‐HDAC5 or GFP alone. GFP and GFP‐HDAC5 were detected by immunoblotting with an anti‐GFP antibody (Roche, 11814460001).
**Figure S2.** Selective activation of PKA in ARVM using N^6^‐benzoyl cAMP. Troponin I (TnI) phosphorylation was used as a readout of PKA activity in ARVM treated with isoprenaline (ISO) for 10 minutes, or with N^6^‐benzoyl cAMP (BNZ), 8‐CPT‐2′‐O‐Me‐cAMP (CPT), or vehicle control (CON) for 30 minutes. A, Five hundred micromoles per liter BNZ induced a similar increase in TnI phosphorylation as 10 nmol/L ISO. B, BNZ treatment increased TnI phosphorylation, whereas equivalent concentrations of CPT had no effect. C, Ten nanomoles per liter ISO and 500 μmol/L BNZ reduced the phosphorylation of HDAC5 at all 3 sites, whereas 500 μmol/L CPT had no effect.
**Figure S3.** Variability associated with measurement of GFP‐HDAC5 nuclear and cytoplasmic fluorescence intensities in live ARVM. A, An ARVM expressing GFP‐HDAC5 was imaged as described in the Methods section. Five sequential z‐sections from an individual cell are shown. The fluorescent signal from GFP‐HDAC5 was used to define the cell boundary (solid white line), the nuclear boundaries (dashed red and yellow lines), and example regions of interest within the cytoplasm (dashed white lines). Scale bar is 10 μm. B, Measurements of the nuclear fluorescence can vary markedly, depending on the focal plane and the nucleus chosen for quantification. Graph shows average fluorescence intensity (*F*) measurements from 3 binucleated cells. Measurements were obtained from 3 sequential z‐sections per nucleus (red and yellow data points for Cell 1 correspond to the red and yellow regions of interest in A). C, There is variability associated with sampling small regions of interest within the cytoplasm. Graph shows *F* measurements for 8 regions of interest per z‐section from sequential z‐sections of the cell shown in A.
**Figure S4.** B55α associates with GFP‐HDAC5. A, Protein lysates (5–15 μL) from ARVM were separated by SDS‐PAGE and the resulting Western blot probed with a mouse monoclonal antibody raised against rat B55α (Santa Cruz sc‐81606). B, Protein lysates from ARVM expressing GFP, GFP‐HDAC5, or neither were immunoprecipitated using a GFP‐Trap kit (Chromotek, gta‐20). Immunoprecipitates were separated on SDS‐PAGE gels and the resulting Western blots probed with antibodies against GFP, B55α, B56α, and B56^δ^.
**Figure S5.** Isoproterenol increases the association between endogenous HDAC5 and B55α. HDAC5‐containing complexes were immunoprecipitated from NRVM cell lysates following treatment with 1 μmol/L isoprenaline (ISO) or vehicle (VEH) for 60 minutes and the resulting Western blots probed for HDAC5 and B55α. Representative Western blots and grouped data from 3 independent experiments. Unpaired *t* test. *P<0.05 vs VEH.
**Figure S6.** Subcellular distribution of B55α in ARVM. ARVM were fractionated into Triton X‐100‐soluble (SOL) and ‐insoluble (INSOL) fractions via centrifugation following treatment with 10 nmol/L isoproterenol (ISO) or vehicle control (CON) for 45 minutes. Representative Western blots and quantitative data from 4 independent experiments. In each experiment, the B55α signal in CON and ISO fractions was normalized to the signal in the respective inputs. No significant differences were detected by unpaired *t* test. The GAPDH and histone 2B blots are the same as those that appear in Figure 2D as the same Western blot was probed for B55α.
**Figure S7.** ISO had no effect on S279 and S498 phosphorylation in NRVM. NRVM expressing GFPHDAC5 were transfected with scrambled (Scr) or *PPP2R2A* (B55α) siRNAs for 48 hours prior to treatment with 1 μmol/L ISO or CON for 60 minutes. Grouped data from 4 independent experiments. No significant differences were detected by 2‐way ANOVA.
**Figure S8.** Quantification of GFP‐HDAC5‐containing puncta in ARVM treated with 10 nmol/L ISO. A, Screenshots from Fiji analysis of GFP‐HDAC5‐containing puncta in live ARVM at baseline and 45 minutes after the addition of 10 nmol/L isoprenaline (ISO). B and C, Quantification of 0.1 to 10 μm^2^ objects at baseline (B) and following treatment with vehicle (VEH) or isoprenaline (ISO) for 45 minutes (C). n=25 to 27 cells from 2 hearts per group. The median and interquartile range are shown. Mann–Whitney tests; ns, not significant.
**Figure S9.** Examples of mislocalized GFP‐HDAC5 in ARVM. Maximum intensity projections of ARVM transduced with adenoviruses to express GFP‐HDAC5. Z‐stacks were acquired as described in Methods. A, Examples of cells that were included in the analysis of GFP‐HDAC5 nucleocytoplasmic shuttling. B, Examples of cells that were excluded from the postimaging analysis of GFP‐HDAC5 nucleocytoplasmic shuttling because of the mislocalization of heterologously expressed GFP‐HDAC5 at baseline. Scale bar is 20 μm.
