# Supplementary material for: β‐Adrenergic Stimulation Induces Histone Deacetylase 5 (HDAC5) Nuclear Accumulation in Cardiomyocytes by B55α‐PP2A‐Mediated Dephosphorylation
Source: J Am Heart Assoc. 2017 Mar 25;6(4):e004861. doi: 10.1161/JAHA.116.004861 (PMC5533001; doi:10.1161/JAHA.116.004861)
Supplement: Supplementary file 1 — Data S1. Supplemental methods. Figure S1. Specificity of antibodies for pS259, pS279, and pS498 in HDAC5. A, Region of human HDAC4 used to generate an antibody against phosphorylated S266 (Walkinshaw et al, 2013) and sequence alignment showing 100% amino acid identity with human HDAC5. The conserved PKA phosphorylation site (S266 in HDAC4; S279 in HDAC5) is underlined. B, Cell lysates from ARVM expressing GFP, wildtype (WT) GFP‐HDAC5, or nonphosphorylatable (S259/498A; S279A) variants of GFP‐HDAC5 were separated by SDS‐PAGE and analyzed by Western blotting. The pS266 HDAC4 polyclonal rabbit antibody detected a band of the correct molecular weight in cells expressing WT and S259/498A GFP‐HDAC5, but not in cells expressing S279A GFP‐HDAC5 or GFP alone, demonstrating cross‐reactivity with pS279 in HDAC5. Similarly, commercially available antibodies for pS259 (Cell Signaling 3443) and pS498 (Abcam 47283) detected bands of the correct molecular weights in cells expressing WT and S279A GFP‐HDAC5, but not in cells expressing S259/498A GFP‐HDAC5 or GFP alone. GFP and GFP‐HDAC5 were detected by immunoblotting with an anti‐GFP antibody (Roche, 11814460001). Figure S2. Selective activation of PKA in ARVM using N6‐benzoyl cAMP. Troponin I (TnI) phosphorylation was used as a readout of PKA activity in ARVM treated with isoprenaline (ISO) for 10 minutes, or with N6‐benzoyl cAMP (BNZ), 8‐CPT‐2′‐O‐Me‐cAMP (CPT), or vehicle control (CON) for 30 minutes. A, Five hundred micromoles per liter BNZ induced a similar increase in TnI phosphorylation as 10 nmol/L ISO. B, BNZ treatment increased TnI phosphorylation, whereas equivalent concentrations of CPT had no effect. C, Ten nanomoles per liter ISO and 500 μmol/L BNZ reduced the phosphorylation of HDAC5 at all 3 sites, whereas 500 μmol/L CPT had no effect. Figure S3. Variability associated with measurement of GFP‐HDAC5 nuclear and cytoplasmic fluorescence intensities in live ARVM. A, An ARVM expressing GFP‐HDAC5 was imaged as described in [file JAH3-6-e004861-s001.pdf]

SUPPLEMENTAL MATERIAL

Data S1.

SUPPLEMENTAL METHODS

Materials

The following reagents were used: laminin (Sigma L2020), M199 (Gibco 22350-029), DMEM (Gibco 11995-065), foetal bovine serum (Gibco 10099-141), carnitine (Sigma C9500), creatine (Sigma C0780), taurine (Sigma T0625), isoprenaline (ISO; Sigma I5627), CGP-20712A (CGP; Sigma C231), ICI 118,551 (ICI; Sigma I127), N^6-benzoyl cAMP (BNZ; Calbiochem 116802), 8-CPT-2'-O-Me-cAMP (CPT; Calbiochem 116833), H89 (Calbiochem, 371962), okadaic acid (OKA; Calbiochem 459620), EDTA-free protease inhibitor cocktail (Roche 11873580001), phosphatase inhibitor cocktail 3 (Sigma P0044). The adenoviral vectors used to express WT GFP-HDAC5, S259/498A GFP-HDAC5 and the MEF2-luciferase reporter were kind gifts from Dr Timothy McKinsey (University of Colorado, USA). The S279A GFP-HDAC5 adenoviral vector was a kind gift from Dr Julie Bossuyt (UC Davis, USA). Adult male Wistar rats (300-324 g) were from Harlan Laboratories (United Kingdom) and Sprague-Dawley rats (1-2 day-old neonates) were from Monash Animal Research Platform (Melbourne, Australia).

ARVM isolation, culture and adenoviral transduction

ARVM were isolated and plated on laminated 6-well culture plates as previously described.[1] Two hours post-plating, the culture medium was replaced with modified M199 medium (M199 medium supplemented with 2 mM L-carnitine, 5 mM creatine, 5 mM taurine and penicillin/streptomycin) containing the appropriate multiplicity of infection (MOI) of adenovirus. The following MOI were used to express GFP or GFP-tagged HDAC5 variants: 3 pfu/cell GFP, 3 pfu/cell WT GFP-HDAC5; 30 pfu/cell S259/498A GFP-HDAC5; 10 pfu/cell S279A GFP-HDAC5. Transduced ARVM were maintained in culture for two days prior to experimentation. For the MEF2-luciferase reporter assay, ARVM were transduced with adenoviral vectors for GFP or GFP-HDAC5 variants as above and with 500 pfu/cell MEF2-luciferase adenovirus 18 hours later. ARVM were cultured for a further 24 hours prior to experimentation.

MEF2 reporter assay

ARVM expressing the GFP-HDAC5 variants or GFP alone and transduced with the MEF2-luciferase reporter (see above) were washed with cold PBS and lysed for 3 minutes on ice in PBS containing 1% Triton X-100, EDTA-free protease inhibitor cocktail and phosphatase inhibitor cocktail 3. The protein concentration of cell lysates was determined by Bradford assay using Protein Assay Dye Reagent (Bio-Rad, 500-0006). 25 μg total protein was incubated with 70 μL ONE-Glo luciferase substrate (Promega, E6110) and the luminescence measured using a GloMax 20/20 Luminometer (Promega) after 10 minutes. Luminescence measurements were normalized to an internal control (ARVM expressing the MEF2-luciferase reporter in the absence of GFP-HDAC5 or GFP) to allow data to be pooled from two independent experiments. Lysates were run on SDS-PAGE gels and the resulting Western blots probed with an anti-GFP antibody to verify that the GFP-HDAC5 variants were expressed to similar levels.

Western blotting

ARVM plated at equivalent densities in 6-well cell culture plates were lysed in 150 μL Laemmli buffer. Proteins were separated by SDS-PAGE and transferred to PVDF membranes for Western blotting. Replicate membranes were probed with antibodies against phosphorylated and total protein species. The following antibodies were used to detect proteins of interest: phospho-S259 HDAC5 (Cell Signaling 3443; 1:1000), phospho-S498 HDAC5 (Abcam 47283; 1:1000), GFP (Roche 11814460001; 1:5000), HDAC5 (Cell Signaling 20458; 1:1000), phospho-S22/23 TnI (Cell Signaling 4004; 1:1000), TnI (Cell Signaling 2002; 1:2000), B55α (Santa Cruz sc-81606; 1:500), B56α (BD Transduction 610615; 1:500), B56δ (Bethyl A301-100A; 1:1000), $PP2A_A$ (Santa Cruz sc-74580; 1:1000), $PP2A_C$ (Cell Signaling 2038; 1:1000). The phospho-S279 HDAC5 antibody (raised against phospho-S266 HDAC4) was a kind gift from Dr Xiang-Jiao Yang (McGill University, Canada). The normalization by sum method was used to group data from biological replicates.[2]

Subcellular fractionation

ARVM were fractionated using a method adapted from Snabaitis *et al*.[3] Briefly, ARVM were lysed in lysis buffer (50 mM Tris-HCl pH 7.5, 5 mM EGTA, 2 mM EDTA, 100 mM NaF, 1% Triton X-100, EDTA-free protease inhibitor tablet) on ice for 5 minutes then centrifuged at 14,000 *g* at 4°C for 30 minutes. The supernatant (containing cytosolic proteins such as GAPDH; "soluble fraction") was collected, and the pellet (containing nuclear proteins such as histone 2B; "insoluble fraction") was resuspended in Laemmli buffer. Samples were resolved on 4-20% SDS-PAGE gels for subsequent Western blotting.

Co-immunoprecipitation of GFP-HDAC5 with PP2A subunits

ARVM were washed in cold PBS and lysed in a high salt lysis buffer (20 mM HEPES-KOH pH 7.4, 250 mM NaCl, 0.11 M KOAc, 2 mM $MgCl_2$, 1 μM $ZnCl_2$, 1 μM $CaCl_2$, 0.5% Triton X-100, 0.1% Tween-20, 500 units/mL Benzonase nuclease (Sigma E1014), EDTA-free protease inhibitor tablet, phosphatase inhibitor cocktail 3) on ice for 3 minutes, then at room temperature for 10 minutes to activate the DNase, as previously described.[4] Cells were scraped and samples vortexed before being incubated on ice for a further 10 minutes. Whole cell lysates were centrifuged at 8,000 *g* at 4°C for 10 minutes, and the NaCl concentration of the resulting supernatant adjusted to 150 mM with NaCl-free lysis buffer. 750 μg of each sample was incubated with uncoupled agarose beads (Chromotek bab-20) for 1 hour at 4°C. The beads were pelleted and the cleared supernatants immunoprecipitated at 4°C overnight using a GFP-Trap A kit (Chromotek gta-20). The next day, immunoprecipitates were washed and resuspended in Laemmli buffer for SDS-PAGE, as previously described.[5]

Detection of endogenous HDAC5-B55α complex

NRVM were isolated from 1-2 day old Sprague Dawley rat pups as previously described[6] and plated in DMEM containing 10% foetal bovine serum and penicillin/streptomycin at a density of $3.5x10^6$ cells per 10 cm cell culture dish. The day after plating, the media was replaced with maintenance media (4:1 DMEM:M199) and the cells cultured for a further 24 hours prior to treatment with 1 μM ISO or vehicle for 60 minutes. Cells were washed in cold PBS and lysed in lysis buffer (30 mM HEPES, 150 mM NaCl, 2 mM $MgCl_2$, 2% Triton X-100, 50 mM NaF, 0.2 mM Na_3VO_4, 1 mM PMSF, 5 μM Pepstatin A) on ice for 10 minutes. Lysates were centrifuged at 8,000 *g* at 4°C for 10 minutes and 500 μg of the resulting supernatant incubated with Protein A Sepharose CL-4B beads (GE Healthcare, 17-0780-01) for 1 hour at 4°C. The beads were pelleted and the cleared supernatants incubated with HDAC5 antibody (Cell Signaling 20458; 1:70) or water (no antibody control) with gentle rocking at 4°C overnight. The next day,

Protein A beads were added and the samples rocked at 4°C for 1 hour. The beads were washed four times in lysis buffer and then resuspended in Laemmli buffer for SDS-PAGE.

siRNA knockdown of B55α in NRVM

NRVM were isolated and plated in 6-well cell culture plates at a density of 350,000 cells/well as described above. The day after plating, NRVM were transduced with WT GFP-HDAC5 adenovirus and incubated in serum-free DMEM overnight. NRVM were transfected with 10 nM *PPP2R2A* siRNA (Dharmacon, L-047957-00-0005) or a non-targeting siRNA pool (Dharmacon, D-001810-10-05) using Lipofectamine RNAiMAX Transfection Reagent (Invitrogen, 13778-150), according to the manufacturer's instructions. Transfected cells were maintained in serum-free DMEM at 37°C, 5% CO_2 for 48 hours prior to stimulation with 1 μM ISO or vehicle for 60 minutes and subsequent lysis for Western blotting.

Quantification of GFP-HDAC5 nucleo-cytoplasmic shuttling

ARVM were isolated, plated in laminated 35 mm imaging dishes (Ibidi, 81156) and transduced with adenoviruses as described above. The day after plating, ARVM were incubated with 0.1 μM Cell Tracker Orange CMRA Dye (Molecular Probes, C34551) for 15 minutes at 37°C, 5% CO_2 and then incubated in fresh modified M199 media overnight. On the day of imaging, cells were imaged using a Nikon Ti-E (inverted) microscope equipped with a Yokogawa spinning disk and a Neo 5.5 sCMOS camera (Andor). A 60x/1.40 NA Plan Apo λ oil objective was used. Images were acquired using NIS Elements AR 4.2 software. Cells were maintained at 37°C, 5% CO_2 throughout the experiment via a CO_2 chamber and a temperature-regulated Perspex box which housed the microscope stage and turret.

The Cell Tracker signal was used to select ARVM for imaging to avoid unnecessary excitation of the GFP fluorophores and to prevent possible bias during the selection of cells based on the basal distribution of GFP-HDAC5. ARVM were not selected for imaging if they satisfied one or more of the following exclusion criteria: 1) cell is not adhered to the bottom of the dish; 2) cell is twitching/moving; 3) cell is balled up or is starting to ball up at one or both ends; 4) cell is overlapping another cell or is close to floating cells (as this interfered with thresholding of the Cell Tracker Orange signal for generation of a binary layer corresponding to the whole cell). After the XY coordinates and perfect focusing system (PFS) settings for 12-15 cells had been set, z-stacks spanning 36 μm (1.5 μm steps) were acquired using the 488 nm laser line to capture the baseline GFP-HDAC5 signal for each cell. Cells were treated with vehicle control or 10 nM ISO and GFP z-stacks repeated 15 minutes post-treatment. Thirty-five minutes post-treatment, 1 μM DRAQ5 (Biostatus, DR50050) was added to the cells to label the nuclei. Ten minutes after the addition of DRAQ5 (i.e. 45 minutes after the addition of ISO/vehicle), each cell was imaged using the 488 nm, 561 nm and 640 nm laser lines to excite the GFP, Cell Tracker and DRAQ5 fluorophores, respectively.

All imaged cells were quantified using NIS Elements AR 4.2 software unless they met one of the following exclusion criteria: 1) cell does not express GFP-HDAC5, 2) z-stack does not cover entire cell volume at one or more time points; 3) cell is rotated in XY plane in one or more time points (rotation is complicated to correct in volume analysis); 4) cell shrinks over the time course of the experiment (as this interfered with the application of binary layers across time points); 5) cell disappears from field of view over the course of the experiment; 6) the GFP signal is saturated in one or more time points; 7) GFP-HDAC5 is clearly mislocalized at baseline (see Fig. S9). Using thresholding, the Cell Tracker and DRAQ5 signals from the final time point were used to generate binary volumes corresponding to the whole cell and nuclei, respectively. Subtraction of the nuclear binary volume from the whole cell binary volume generated a third binary volume corresponding to the cytoplasm. The binary volumes were then

used to determine average GFP fluorescence intensities in the nuclear, cytoplasmic and whole cell volumes at each time point.

Quantification of fluorescent puncta

The number of puncta was quantified using Fiji imaging analysis software.[7] For each cell, the background was subtracted using a rolling ball radius of 50.0 pixels. A single z-section that approximately bisected at least one of the nuclei was selected from the baseline GFP z-stack and the threshold adjusted by altering the minimum intensity value until there was a light ‘salt and pepper’ effect within the cell (see Fig. S8A). The number of puncta in the resulting binary image was quantified by counting the number of 0.1–10 μm^2 objects using the ‘Analyze Particles’ function. Any artifacts (e.g. GFP fluorescence in the nuclei) were subtracted from the total object count. The same conditions were then used to quantify the number of puncta in the corresponding z-section from the 45 minute GFP z-stack. The entire analysis was performed blinded to treatment group and z-stacks from the 45 minute time point were not viewed by the user until the analysis was complete to eliminate possible bias in application of the threshold to the baseline z-section image.

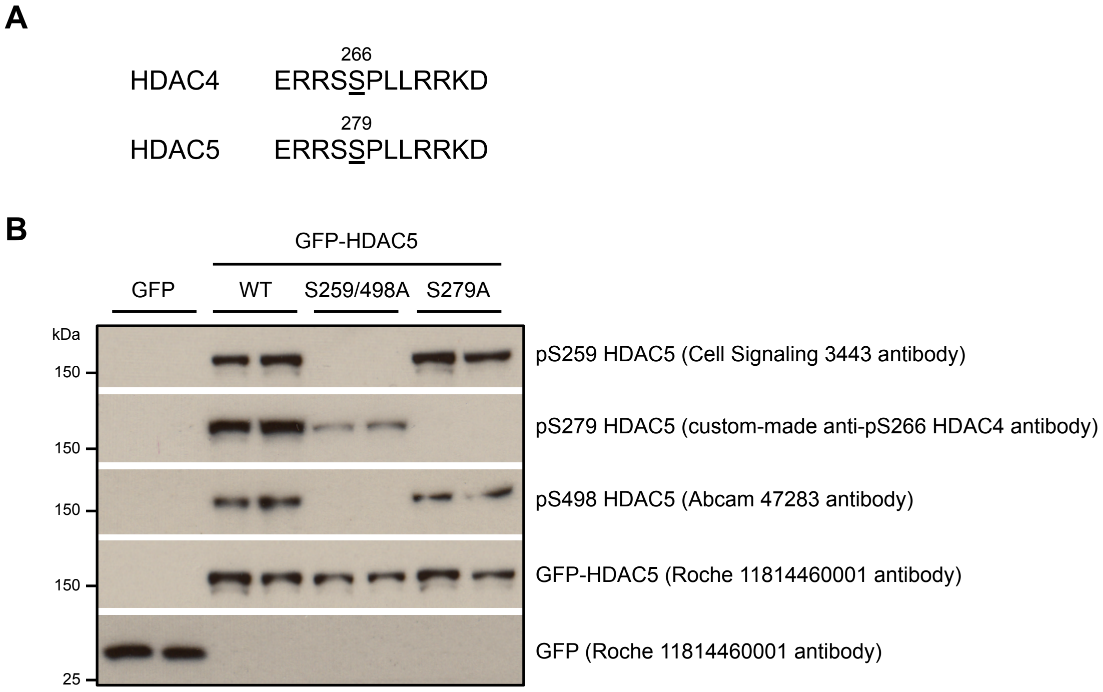

Figure S1. Specificity of antibodies for pS259, pS279 and pS498 in HDAC5. A, Region of human HDAC4 used to generate an antibody against phosphorylated S266 (Walkinshaw *et al.*, 2013) and sequence alignment showing 100% amino acid identity with human HDAC5. The conserved PKA phosphorylation site (S266 in HDAC4; S279 in HDAC5) is underlined. **B,** Cell lysates from ARVM expressing GFP, wildtype (WT) GFP-HDAC5, or non-phosphorylatable (S259/498A; S279A) variants of GFP-HDAC5 were separated by SDS-PAGE and analysed by Western blotting. The pS266 HDAC4 polyclonal rabbit antibody detected a band of the correct molecular weight in cells expressing WT and S259/498A GFP-HDAC5, but not in cells expressing S279A GFP-HDAC5 or GFP alone, demonstrating cross-reactivity with pS279 in HDAC5. Similarly, commercially available antibodies for pS259 (Cell Signaling 3443) and pS498 (Abcam 47283) detected bands of the correct molecular weights in cells expressing WT and S279A GFP-HDAC5, but not in cells expressing S259/498A GFP-HDAC5 or GFP alone. GFP and GFP-HDAC5 were detected by immunoblotting with an anti-GFP antibody (Roche, 11814460001).

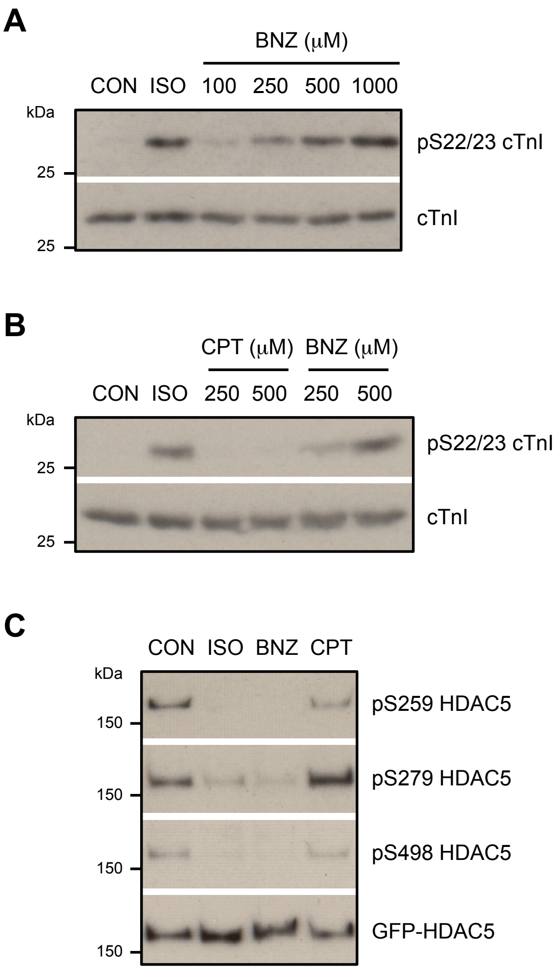

Figure S2. Selective activation of PKA in ARVM using N^6-benzoyl cAMP. Troponin I (TnI) phosphorylation was used as a readout of PKA activity in ARVM treated with isoprenaline (ISO) for 10 min, or with N^6-benzoyl cAMP (BNZ), 8-CPT-2'-O-Me-cAMP (CPT) or vehicle control (CON) for 30 min. **A,** 500 μM BNZ induced a similar increase in TnI phosphorylation as 10 nM ISO. **B,** BNZ treatment increased TnI phosphorylation, whereas equivalent concentrations of CPT had no effect. **C,** 10 nM ISO and 500 μM BNZ reduced the phosphorylation of HDAC5 at all three sites, whereas 500 μM CPT had no effect.

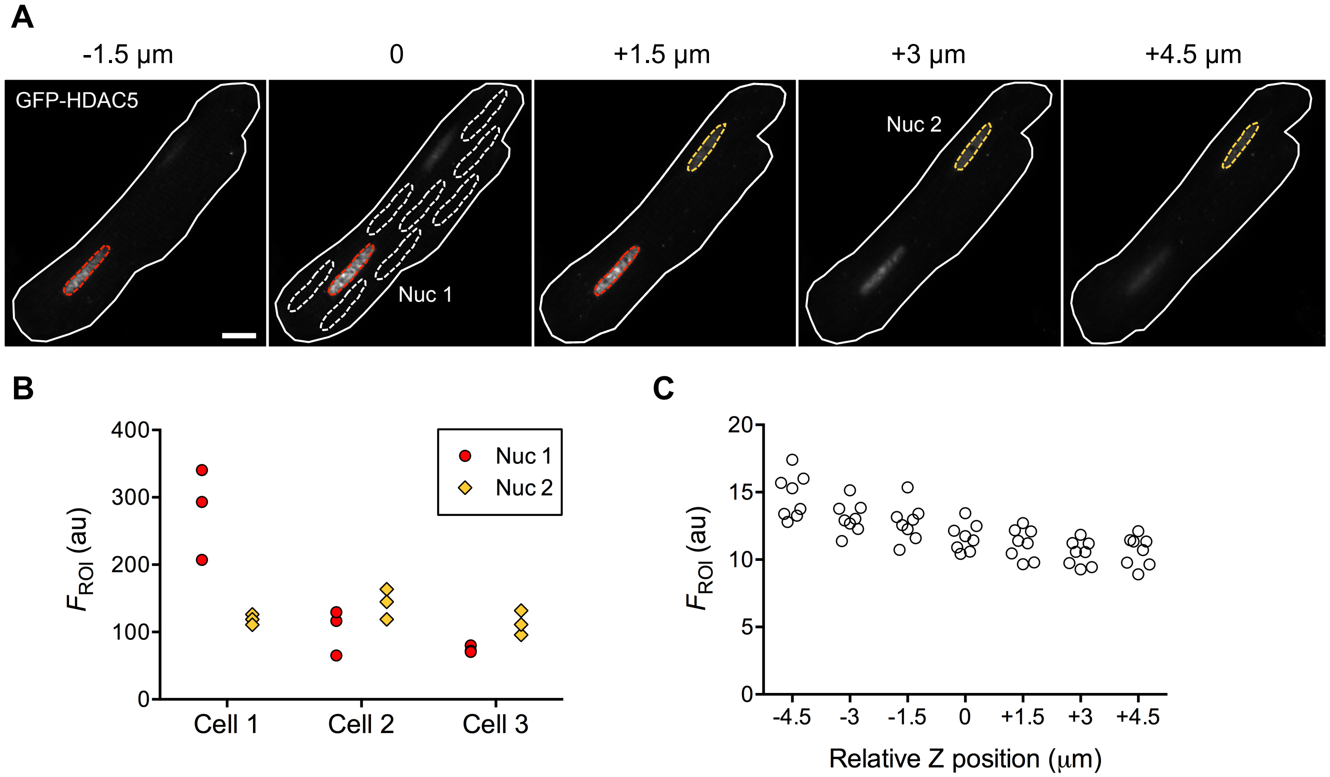

Figure S3. Variability associated with measurement of GFP-HDAC5 nuclear and cytoplasmic fluorescence intensities in live ARVM. A, An ARVM expressing GFP-HDAC5 was imaged as described in Materials and Methods. Five sequential z-sections from an individual cell are shown. The fluorescent signal from GFP-HDAC5 was used to define the cell boundary (solid white line), the nuclear boundaries (dashed red and yellow lines) and example regions of interest (ROI) within the cytoplasm (dashed white lines). Scale bar is 10 µm. **B,** Measurements of the nuclear fluorescence can vary markedly, depending on the focal plane and the nucleus chosen for quantification. Graph shows average fluorescence intensity (*F*) measurements from three binucleated cells. Measurements were obtained from three sequential z-sections per nucleus (red and yellow data points for Cell 1 correspond to the red and yellow ROI in panel A). **C,** There is variability associated with sampling small ROI within the cytoplasm. Graph shows *F* measurements for eight ROI per z-section from sequential z-sections of the cell shown in panel A.

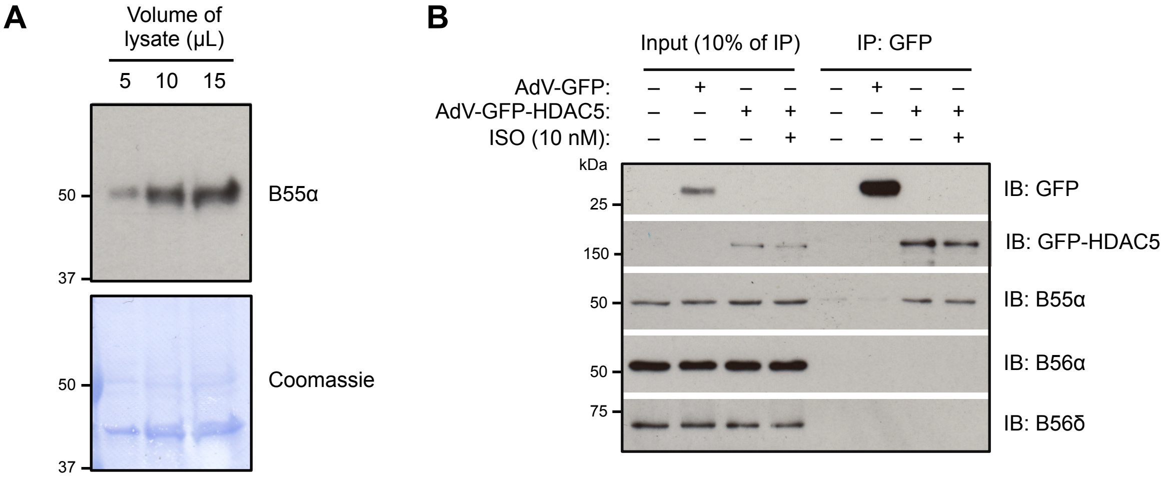

Figure S4. B55α associates with GFP-HDAC5. A, Protein lysates (5-15 μL) from ARVM were separated by SDS-PAGE and the resulting Western blot probed with a mouse monoclonal antibody raised against rat B55α (Santa Cruz sc-81606). **B,** Protein lysates from ARVM expressing GFP, GFP-HDAC5 or neither were immunoprecipitated using a GFP-Trap kit (Chromotek, gta-20). Immunoprecipitates were separated on SDS-PAGE gels and the resulting Western blots probed with antibodies against GFP, B55α, B56α and B56δ.

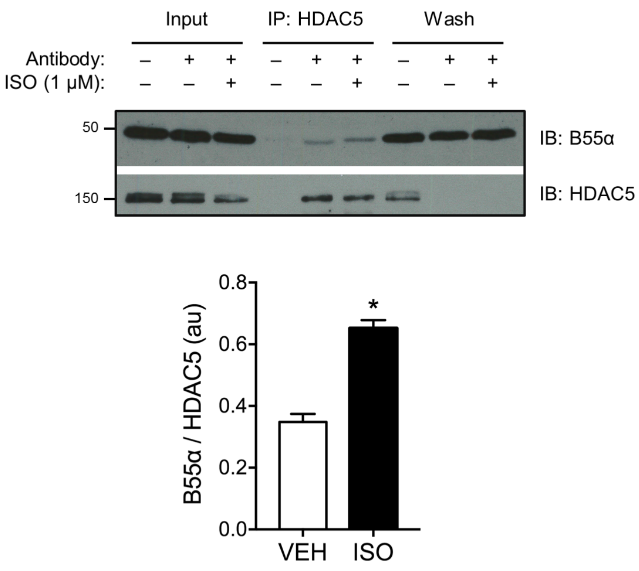

Figure S5. ISO increases the association between endogenous HDAC5 and B55α. HDAC5-containing complexes were immunoprecipitated from NRVM cell lysates following treatment with 1 μM isoprenaline (ISO) or vehicle (VEH) for 60 minutes and the resulting Western blots probed for HDAC5 and B55α. Representative Western blots and grouped data from three independent experiments. Unpaired *t*-test. * $P<0.05$ vs VEH.

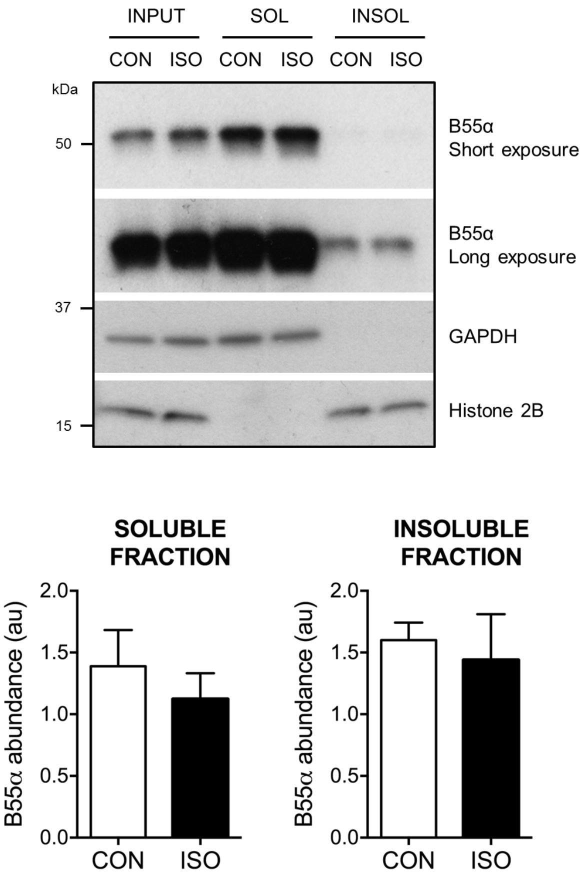

Figure S6. Subcellular distribution of B55α in ARVM. ARVM were fractionated into Triton X-100-soluble (SOL) and -insoluble (INSOL) fractions via centrifugation following treatment with 10 nM isoprenaline (ISO) or vehicle control (CON) for 45 minutes. Representative Western blots and quantitative data from four independent experiments. In each experiment, the B55α signal in CON and ISO fractions was normalised to the signal in the respective inputs. No significant differences were detected by unpaired *t*-test. The GAPDH and histone 2B blots are the same as those that appear in Fig. 2D as the same Western blot was probed for B55α.

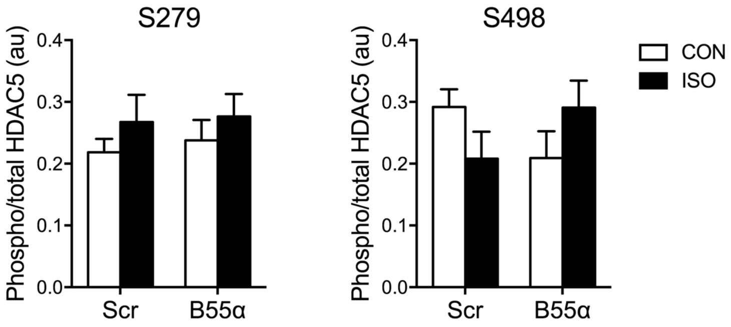

Figure S7. ISO had no effect on S279 and S498 phosphorylation in NRVM. NRVM expressing GFP-HDAC5 were transfected with scrambled (Scr) or *PPP2R2A* (B55α) siRNAs for 48 hours prior to treatment with 1 μM ISO or CON for 60 minutes. Grouped data from four independent experiments. No significant differences were detected by two-way ANOVA.

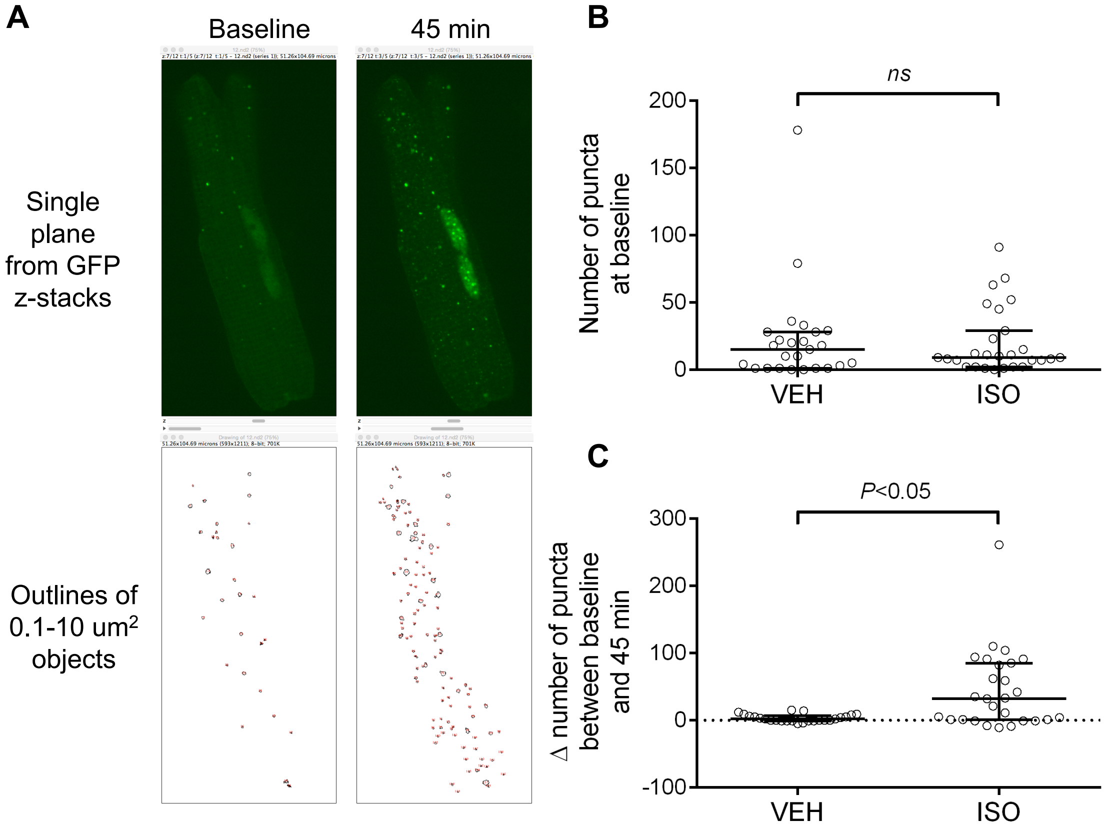

Figure S8. Quantification of GFP-HDAC5-containing puncta in ARVM treated with 10 nM ISO. A, Screenshots from Fiji analysis of GFP-HDAC5-containing puncta in live ARVM at baseline and 45 minutes after the addition of 10 nM isoprenaline (ISO). **B, C,** Quantification of 0.1-10 μm^2 objects at baseline (**B**) and following treatment with vehicle (VEH) or isoprenaline (ISO) for 45 minutes (**C**). *n*=25-27 cells from two hearts per group. The median and interquartile range are shown. Mann-Whitney tests; ns: not significant.

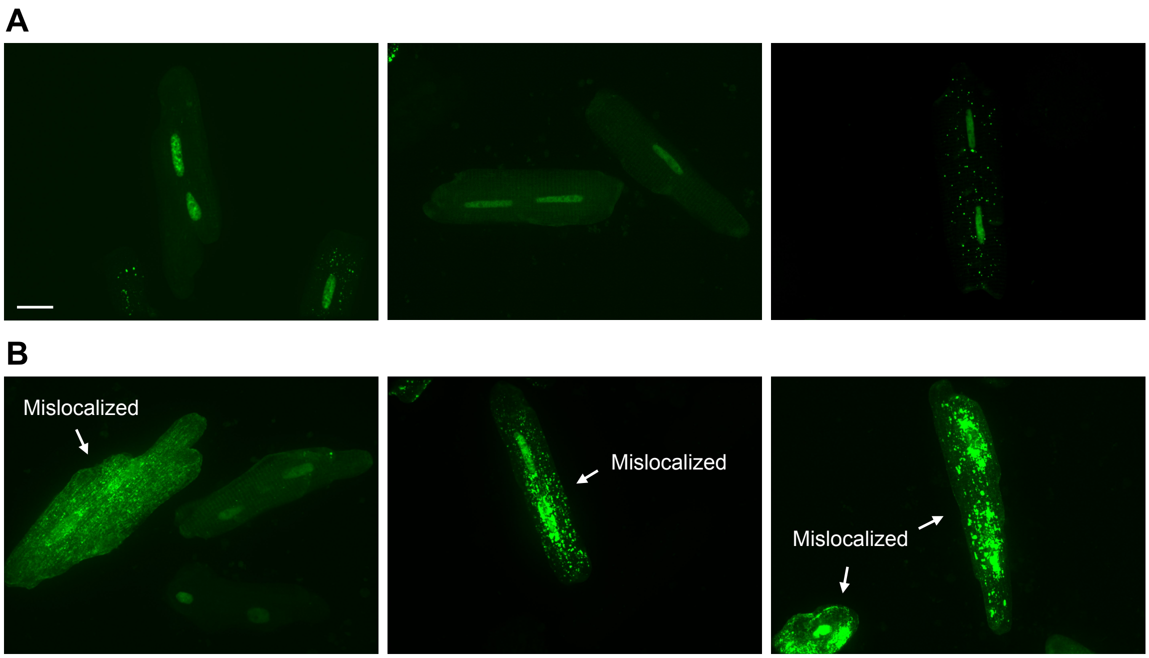

Figure S9. Examples of mislocalized GFP-HDAC5 in ARVM. Maximum intensity projections of ARVM transduced with adenoviruses to express GFP-HDAC5. Z-stacks were acquired as described in Methods. **A,** Examples of cells that were included in the analysis of GFP-HDAC5 nucleo-cytoplasmic shuttling. **B,** Examples of cells that were excluded from the post-imaging analysis of GFP-HDAC5 nucleo-cytoplasmic shuttling due to the mislocalization of heterologously-expressed GFP-HDAC5 at baseline. Scale bar is 20 μm.

SUPPLEMENTAL REFERENCES:

1. Snabaitis AK, Muntendorf A, Wieland T and Avkiran M. Regulation of the extracellular signal-regulated kinase pathway in adult myocardium: differential roles of G(q/11), Gi and G(12/13) proteins in signalling by alpha1-adrenergic, endothelin-1 and thrombin-sensitive protease-activated receptors. *Cell Signal*. 2005;17:655-64.
2. Degasperi A, Birtwistle MR, Volinsky N, Rauch J, Kolch W and Kholodenko BN. Evaluating strategies to normalise biological replicates of Western blot data. *PloS One*. 2014;9:e87293.
3. Snabaitis AK, D'Mello R, Dashnyam S and Avkiran M. A novel role for protein phosphatase 2A in receptor-mediated regulation of the cardiac sarcolemmal Na^+/H^+ exchanger NHE1. *J Biol Chem*. 2006;281:20252-20262.
4. Joshi P, Greco TM, Guise AJ, Luo Y, Yu F, Nesvizhskii AI and Cristea IM. The functional interactome landscape of the human histone deacetylase family. *Mol Syst Biol*. 2013;9:672.
5. Stathopoulou K, Cuello F, Candasamy AJ, Kemp EM, Ehler E, Haworth RS and Avkiran M. Four-and-a-half LIM domains proteins are novel regulators of the protein kinase D pathway in cardiac myocytes. *Biochem J*. 2014;457:451-61.
6. Sapra G, Tham YK, Cemerlang N, Matsumoto A, Kiriazis H, Bernardo BC, Henstridge DC, Ooi JY, Pretorius L, Boey EJ, Lim L, Sadoshima J, Meikle PJ, Mellet NA, Woodcock EA, Marasco S, Ueyama T, Du XJ, Febbraio MA and McMullen JR. The small-molecule BGP-15 protects against heart failure and atrial fibrillation in mice. *Nat Commun*. 2014;5:5705.
7. Schindelin J, Arganda-Carreras I, Frise E, Kaynig V, Longair M, Pietzsch T, Preibisch S, Rueden C, Saalfeld S, Schmid B, Tinevez JY, White DJ, Hartenstein V, Eliceiri K, Tomancak P and Cardona A. Fiji: an open-source platform for biological-image analysis. *Nat Methods*. 2012;9:676-82.
